# Consistent role of weak and strong interactions in high- and low-diversity trophic food webs

**DOI:** 10.1038/ncomms11180

**Published:** 2016-04-12

**Authors:** Gabriel Gellner, Kevin S. McCann

**Affiliations:** 1 Department of Environmental Science and Policy, University of California Davis, 1 Shields Avenue, Davis, California 95616, USA; 2 Department of Integrative Biology, University of Guelph, Guelph, Ontario, Canada N1G 2W1

## Abstract

The growing realization of a looming biodiversity crisis has inspired considerable progress in the quest to link biodiversity, structure and ecosystem function. Here we construct a method that bridges low- and high-diversity approaches to food web theory by elucidating the connection between the stability of the basic building block of food webs and the mean stability properties of large random food web networks. Applying this theoretical framework to common food web models reveals two key findings. First, in almost all cases, high-diversity food web models yield a stability relationship between weak and strong interactions that are compatible in every way to simple low-diversity models. And second, the models that generate the recently discovered phenomena of being purely stabilized by increasing interaction strength correspond to the biologically implausible assumption of perfect interaction strength symmetry.

## Abstract (web summary)

High-and low-diversity food webs are thought to differ in their structural stability. Here, the authors use a method that bridges both levels of diversity to show that stability relationships and interaction strength can be consistent between simple and complex trophic networks.

## Graphical abstract

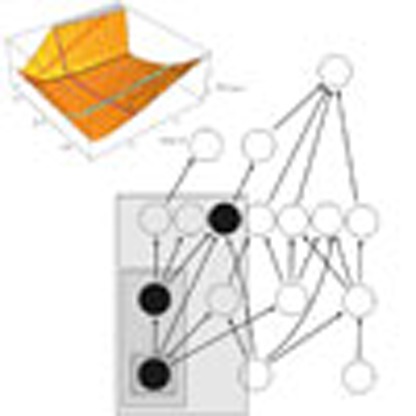

The dynamics of ecosystems include a bewildering number of weak to strong biotic interactions. Global human development has begun to erode this natural complexity making it important that we rapidly assess the structural aspects of food webs that are critical to the stable function of our life-support systems^1^. Importantly, results from simple low-diversity food web models often stand in contrast to results from complex high-diversity food web models on the role that diversity and structure play in mediating the stability of nature's complex networks. Of particular interest is the recent discovery that large predator–prey networks seem to be purely stabilized by increasing interaction strength^2^,^3^; a result that overturns 40 years of ecological research^4^,^5^,^6^,^7^,^8^,^9^.

There exists a long and storied history of research on the relationship between diversity, complexity and stability of whole food webs^10^. Despite this, there has been little attempt to bridge the understanding of stability results from population ecology to whole food webs. This is surprising given that the underlying sub-systems of whole webs are in fact both populations and low-diversity interactions, and so a search for internal consistency seems logical (Fig. 1a–d).

Here we first identify a very general argument for the role of biomass growth and biological lags on the stability of single populations. We then show that this simple rule translates to low-diversity modular interactions, or food web modules^11^, where consumer species growth rates (that is, numerical response) and inherent consumer–resource (C–R) lags dictate an identical stability response. Finally, we consider results from whole food web models where current and historical research finds a mixture of stability responses that seem either partially, or wholly, incongruent with this simple general rule^2^,^3^. We then revisit these results using the elegant mathematical result of Sommers *et al*.^12^ to show that both historical and recent results are singular endpoints that hide a general result that is equivalent to the population and modular results. Indeed, low- and high-diversity food webs generally yield qualitatively similar stability results.

## Results

### General ecological stability relationship

The continuous logistic model can be solved to see that increased population stability scales directly with increased population growth rates such that increased growth rates yield increased stability (that is, *λ=−r*). This well-known result shows that high growth, without a lag, simply stabilizes the population—a small perturbation of a high growth rate population returns rapidly back to the equilibrium. While a simple and intuitive result, population ecologists and mathematicians have long understood that lags tend to induce instability. Ecological systems abound with lags, and so while the continuous logistic is a good example of population growth, it is a poor model for understanding stability since it ignores the fact that populations are replete with lags.

**Figure 1:**
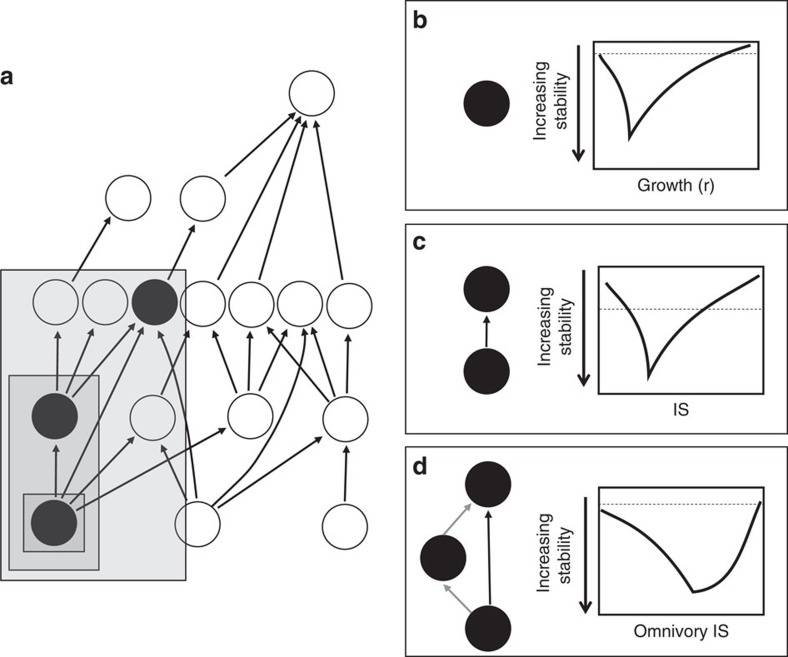
Decomposition of food webs into stability building blocks. Modular theory attempts to decompose complex food webs like in **a** by looking at the stability patterns as interaction strength (IS) is changed of the simpler, sub-web (filled circles), building blocks (grey boxes). A general finding from modular theory is a stability tradeoff for increasing the flux/growth/interaction strength, where we expect an initial stabilization phase as increased energy leads to increased resilience/stability as populations can respond to perturbations more rapidly. Ultimately this same increased energy begins to lead to overshoot, as the populations respond so rapidly, coupled with a lag, that the return to equilibrium is missed. This behaviour occurs for lagged population models (**b**) consumer-resource interactions (**c**) as well as higher order food web modules such as same chain omnivory (**d**).

To rectify this, we next consider a broad set of population growth models that incorporate biological lags (for example, the lagged logistic model) and find that now increasing the intrinsic growth rate leads to a dual response: (i) a stabilizing phase where increases in growth rates increases the equilibrium stability (low *r* phase in Fig. 1b); and (ii) a destabilizing phase where increases in growth rates destabilize the equilibrium (high *r* phase in Fig. 1b). This dual stability response, or ‘checkmark' stability pattern, is universal to a broad set of population models (Supplementary Note 1 and Supplementary Fig. 1).

Mathematically, it can be shown that the switch from stabilization to destabilization in Fig. 1b occurs for the lagged logistic at exactly the point the eigenvalue, *λ*, becomes complex (negative for discrete models; Supplementary Note 1 and Supplementary Fig. 2). From a biological standpoint this mathematical results implies that the destabilizing phase always occurs at precisely the point where the population starts to show overshoot dynamics (that is, population dynamics begin to vary around carrying capacity, *K*, on their approach to the equilibrium). In a simplified sense, this overshoot can be envisioned as the expression of the population lag such that further increases in growth (*r*) operate to only increase overshoot dynamics and further destabilize the dynamics. In many population models this destabilizing influence of increasing growth rates coupled to lags eventually lead to wild oscillations and even chaos^4^,^13^.

To summarize, when a population has lags we find a general checkmark stability pattern whereby increasing growth rate (or net per capita flux into the population) first stabilizes a population but then ultimately destabilizes the population by driving overshoot dynamics that are further excited by increasing per capita flux into the population. We now extend this result to C–R models and low-diversity food web modules.

### Stability pattern in C–Rs models

As with the population model, the C–R interaction is an inherent and ubiquitous sub-system in any food web (Fig. 1c). Following our single population analysis, we start by considering what governs the growth of the consumer in the C–R interaction. In classical Lotka–Volterra models, it is assumed that the biomass growth process is a result of the conversion of resource consumption (for example, *eaRC* for Type I, where *R* is the resource biomass, *C* is the consumer biomass, *e* is conversion and *a* is attack rate). Thus, the C–R analogue of the intrinsic growth rate of a single population is related to interaction strength or, more accurately, the strength of the parameters governing the interaction (that is, *ea* is to the *eaRC* as *r* is to *rR*). Note also that in a C–R interaction, a biological lag occurs implicitly due to the time needed to convert resource biomass into consumer biomass, and therefore, no explicit lag is required.

Figure 1c shows the generic response of increasing *ea* of a Type I C–R model with the standard logistic resource growth. Not surprisingly, the result exactly mirrors the population growth result with a checkmark stability pattern. Here, as before, the destabilizing phase of increasing consumer interaction potential occurs at precisely the point where the population dynamics start to overshoot (Supplementary Note 2 and Supplementary Figs 3–6, for examples, showing identical response across a broad set of C–R models). The checkmark response is clearly related to the transcritical bifurcation (where C becomes feasible) in the stabilizing phase, while the destabilizing phase can often be associated with a Hopf bifurcation. Note, although, that the destabilizing phase only depends on the presence of complex eigenvalues and so really only necessitates that the dynamics show transient oscillatory decay. Finally, this result readily extends to simple low-diversity food web modules where we increase the interaction potential (for example, *ea*) of all interactions simultaneously (Fig. 1d; Supplementary Note 1 and 2). In summary, theoretical results from both population and C–R-based food web modules generically show a checkmark stability response to increases in growth (similarly, interaction strength within the C–R framework). It remains to consider whole webs.

### Scaling up to whole food webs

Forty years ago, Robert May, using community matrices, showed mathematically and numerically that randomly constructed whole webs had local stability properties well approximated for large numbers of species (S) by the following relationship for the maximum eigenvalue (*λ*_max_):


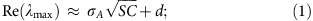


where *C* is connectance, and *σ*_*A*_ is the s. d. of the union of non-zero off-diagonal matrix elements (hereafter referred to as A). Increasing *σ*_*A*_ was argued to be a surrogate for increasing mean interaction strength as each matrix element had a mean of zero and therefore increasing *σ*_*A*_ increased the mean range of values in the community matrix. Finally, *d* is the size of the diagonal or the per capita effects that a species has on themselves (*a*_*ii*_; −1 for May's special case). As May pointed out, for the case of diagonals set at −1 then the system is stable whenever the positive term, 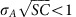. This simple, but elegant, formulation suggests that strengthening interactions (increasing *σ*_*A*_) is purely destabilizing. From this result, we shall hereafter refer to the term, 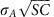 in equation 1, as ‘May's destabilization factor'.

While an interesting approach, this specific relationship holds for matrices of random interactions alone (random mixtures of C–R, competition and mutualism^3^). It also assumes that the off-diagonal pairs, on average, have equal means. Thus, while any off-diagonal pair is different, the property of the whole matrix, on average, produces equal off-diagonal pairs. There are several reasons why this equation may not be appropriate for food webs constructed purely from C–R interactions. In consumptive webs like these, the non-zero off-diagonal elements, *a*_*ij*_, (representing per capita impacts of species *j* on species *i*) will be non-negative for the consumer and negative for the resource. In addition, Lotka–Volterra models find that the positive interaction strength of the resource on the consumer (*a*_*ji*_) should scale with the negative interaction strength of the consumer on the resource (*a*_*ij*_) as *f*=*a*_*ji*_/*a*_*ij*_. For a Type I functional response, *f*=eC*/R* implies that *f* should generally be much less than 1 (note, it would take C* to be considerably greater in biomass than R* for *f* to scale ≥1 and this is empirically known to be rare^14^,^15^). An issue we do not consider is using measures of density rather than energy/biomass. This complicates the meaning of *f* as we lose the nice biological relationship to biomass pyramids. In general, the theory presented here will carry over to using density but it is harder to interpret the meaning of the parameters.

Here to embody the biological interpretation of biomass pyramids (C:R ratios), we choose community matrices with a scaling parameter, *f*. First, to enforce a consumptive food web structure we define two distributions; one corresponding to the negative effect of the consumer on the resource (*A*_*ij*_) and a second for the positive effect the resource has on the consumer (*A*_*ji*_). These distributions are called the half distributions of the community matrix as they correspond to each half of the paired C–R interactions. Specifically, we construct C–R interactions with the negative effect of the consumer on the resource drawn from a ‘half' distribution *A*_*ij*_ with mean *E*[*A*_*ij*_], and the effect of the resource on the consumer drawn from the scaled half distribution, *A*_*ji*_=*fA*_*ij*_, with mean *fE*[*A*_*ij*_]. With this formulation the distribution of non-zero off-diagonal matrix elements (A) is equal to the union of the half distributions {*A*_*ij*_, *fA*_*ij*_}. Notice that this approach does not mean all non-diagonal pairs follow the exact scaling relationship, that is, *fa*_*ji*_≠*a*_*ij*_, but rather it implies that the mean properties of all non-diagonal pairs do (*fE*[*A*_*ij*_]=*E*[*A*_*ij*_]). This approach allows for natural variation between the scaling of each specific C–R pair while maintaining a mean symmetry for the entire matrix.

Amazingly, there is an analytic estimate for the dominant eigenvalue for matrices with similar symmetric structure derived by Sommers *et al*.^12^ for the bivariate normal distribution, and conjectured to hold for more general distributions (unpublished proofs of the general case have recently been proposed^16^,^17^). Building on an estimate by Sommers *et al*.^12^, Allesina & Tang^3^ added the connectance structure of food web models, in an analogous fashion to May, yielding the general stability estimate for C–R networks:


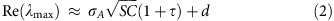


where, again, *σ*_*A*_ is the standard deviation of the whole distribution and *τ* is related to the correlation between the off-diagonals (see Supplementary Note 4 and Supplementary Figs 10–14 for technical details). This estimate for the stability of C–R matrices includes May's destabilizing factor, 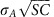, modified by (1+*τ*). Since *τ* ranges from −1 to 0 in predator–prey interaction matrices (Supplementary Note 4), the (1+*τ*) term ranges between 0 and 1. Thus (1+*τ*) is a measure of the damping in equation (2) caused by the structure of the interaction strength distribution (hereafter, we refer to (1+*τ*) as the damping factor). If the damping factor is 0, then the system will be maximally stable, as the influence of May's destabilization factor is removed. On the other hand, if the damping factor is 1, then we have maximum destabilization and a return to equation 1. For intermediate values of the damping factor, we scale the strength at which May's destabilization factor operates.

It is important to recognize that the exact value of *τ* for any community matrix depends on the magnitude of mean interaction strengths, *E*[*A*_*ij*_], which depends on the variation of the half distribution, 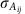, as well as the interaction strength scaling parameter, *f*. The precise relationship between *τ*, *σ*_*A*_ and the scaling parameter, *f*, dictate the stability outcome (equation 2, above). Due to the asymmetry in our whole-web experiments (that is, *f*≠1), we cannot directly employ Allesina and Tang's formula for *τ*, so instead we numerically derive the true dominant eigenvalue and compare this to the heuristic method described. Note that equation 2 still often remains an excellent estimate for the *f*≠1 case (Supplementary Note 4) and is not always outperformed by more recent conjectured formula's that try to include non-symmetric C–R pairs (Supplementary Note 4). In any case, all formula's give qualitatively similar answers so we choose to use the simplest form. In what follows, we use our numerical matrix results and equation 2 as a heuristic guide to explore the stability implications of several common interaction strength experiments.

### Interaction strength and stability

**Figure 2:**
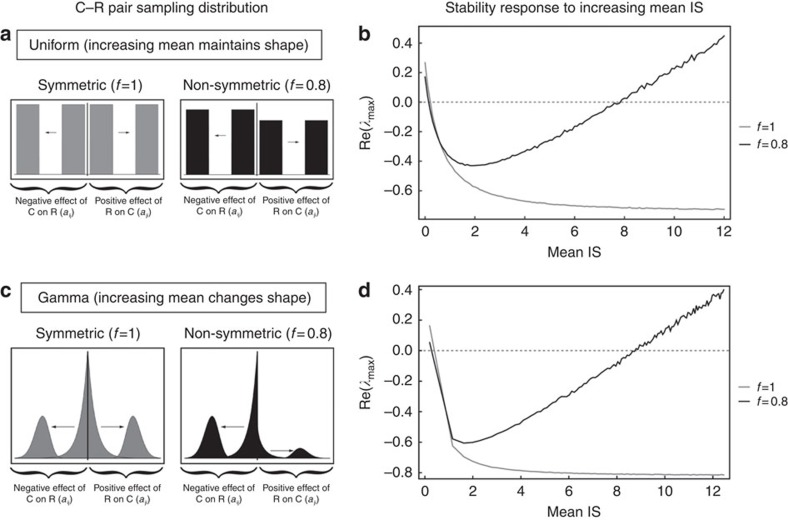
Consistent stability pattern for large random trophic food webs. The role of increasing mean interaction strength (IS) and food web stability exhibits a universal checkmark stability signature (**b**,**d**) as long as the scaling of the consumer–resource (C–R) interaction pairs (*f*) is not exactly 1 (**a**,**c**). This signature does not depend on the shape of the C–R pair interaction distribution changing with mean interaction strength. We show two examples: fixed higher moments (Uniform, **a**), changing higher moments (Gamma, **b**) both have qualitatively identical patterns. For each model the diversity is set as *S*=250, connectance is set as *C*=0.25, with off-diagonal community matrix elements drawn from a uniform distribution Uniform (*r*, *r*+1) with *r* ranging within [0, 10] in steps of 0.1, and a gamma distribution 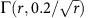 with *r* ranging within [1, 2,480] in steps of 20.0. In both models (**a**,**b**), for each value of *r* we created 100 random network topologies (location of non-zero matrix elements) and then 100 random community matrices and took the mean real part of the dominant eigenvalue as a measure of the stability and plotted this versus the mean of the C–R pair sampling distribution.

The first numerical experiment conducted was to increase the mean of the half distributions (*E*[*A*_*ij*_]) of the C–R matrix pairs while keeping the variance of the half distribution 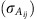 equal (Fig. 2a). This necessarily increases *σ*_*A*_ and May's destabilizing factor, however, the stability response (equation 2) requires that we understand how the damping term is influenced by increases in *σ*_*A*_. It turns out that the qualitative behaviour of the damping response depends entirely on whether *f*=1 or *f*≠1.

Conducting this experiment with a symmetrical interaction scaling parameter (that is, *f*=1), we find that as we increase the average interaction strength (that is., increasing *σ*_*A*_), we initially decrease the damping term, 1+*τ*, until the damping term becomes exactly 0. Note that the *f*=1 symmetry assumption drives the damping term to 0, and does not depend on the distribution used. This monotonic approach of 1+*τ* to 0 means that the destabilizing potential of May's factor (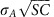) is increasingly muted until we reach maximal stability (determined by the diagonal value *d*). This relationship between 1+*τ* and mean interaction strength drives a pure stabilization result (Fig. 2b; grey curve). Thus, for perfectly symmetrical mean C–R interaction strengths, our first whole-web result is that increasing mean interaction strength is purely stabilizing—an answer inconsistent with population ecology and results from modular food web models.

Importantly, changing *f* from anything but 1 decreases maximum achievable damping and we find that it necessarily approaches a value greater than 0 as mean interaction strength increases. If we now conduct the same experiment with any *f*-value other than 1, then we see from equation 2 that we get a different stability answer. Increasing mean interaction strength again drives 1+*τ* towards some minimal value (but this value is now greater than 0). During this phase, the result operates identically to the *f*=1 case in that increasing interaction strength stabilizes the system by muting May's destabilization term. However, once the 1+*τ* term saturates with increasing interaction strength (that is, increasing *σ*_*A*_), May's growing destabilizing factor (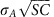) overwhelms equation 2, driving a destabilization phase (Fig. 2b; black curve). The result is the now familiar checkmark stability pattern (Fig. 2b; black curve). In summary, we have shown that extremely complex webs have a stability result identical with population and low-diversity models for all but one degenerate configuration (that is, perfect interaction strength mean symmetry; *f=1*). Although we have concentrated in the examples presented here with the *f*<1 to try and match the most common biological situations^14^,^15^ the relationship holds, as we have generally stated for *f*≠1, see Supplementary Note 7 and Supplementary Fig. 18 for a thorough example where we change both mean interaction strength as well as *f*, both greater and less than 1 and show the full stability surface.

Researchers have also been interested in how the skew of interaction strength distributions influence stability, and modular food web results make the prediction that skews towards strong interactions ought to be destabilizing^6^,^7^. To test, we carried out numerical experiments that changed the shape of the half distribution from one having a bias towards weak interactions into a distribution with a bias towards strong interactions (Fig. 2c). We then tracked the stability results for both symmetrical (*f=*1; Fig. 2d; grey curve) and non-symmetrical (*f*≠1; Fig. 2d; black curve) many species matrices. The non-symmetric case again yields the checkmark stability pattern (Fig. 2d; black curve). This occurs because the s. d. of the whole distribution, *σ*_*A*_, necessarily increases in this experiment, and so May's destabilization factor eventually overwhelms the damping as we change the skew towards strong interaction strengths (Supplementary Notes 5 and 6 and Supplementary Figs 15–17 for further details on how the shape of the distributions interacts with stability).

### The role of realistic network structure

To this point, we have used purely random network structures (lacking any biological constraints). This is arguably an extreme assumption as we have good statistical models for the topology of food webs in nature^18^. To get at this, we redo our above analysis but employing three additional network models: the Cascade^19^, which gives a more realistic link distribution, the Generalized Cascade^20^, which gives a small update to the original Cascade model so that it fits ecological data better, and finally the mostly widely used model of food web topology the Niche model^21^. What is nice about this selection of models is that we have an increasing level of complexity from the purely Random model of May continuing up to the Niche where we are able to keep the same underlying structure of the number of species (*S*) and the connectance (*C*) the same, while changing the underlying structure like the link distribution in a manner that has been shown to fit ecological data well^20^.

**Figure 3:**
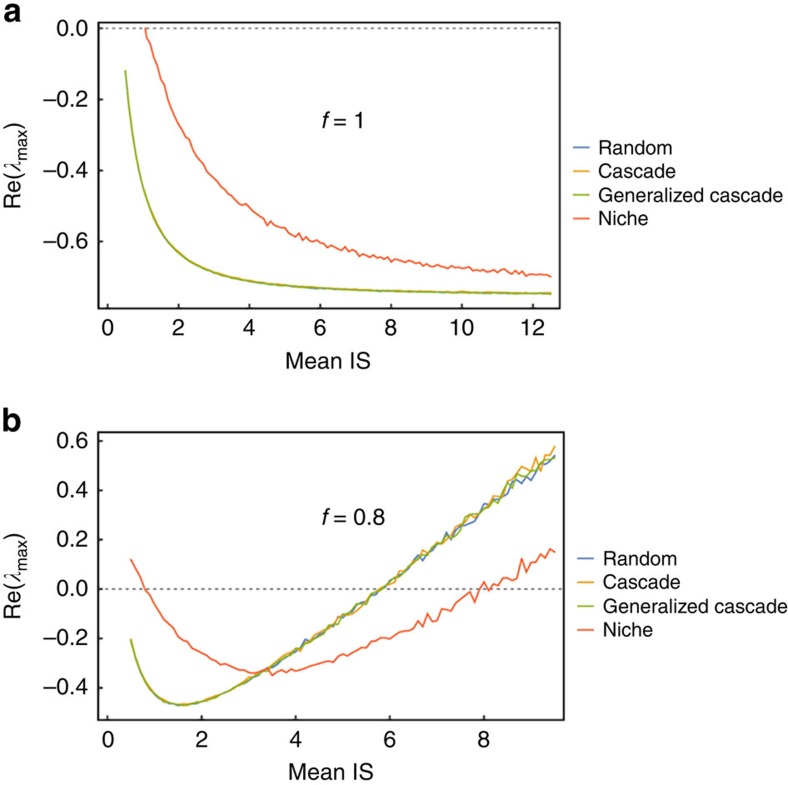
Consistent Stability Pattern for Changing Network Topology. The effect of increasing the realism of the topology of consumer–resource (C–R) interactions for symmetric and asymmetric C–R interaction distributions. Each model has 100 species, with a connectance of 0.3, the interaction strength distribution was the Uniform(r, *r*+1) where *r* ranged from 0 to 15 in increments of 0.1. In **a**, we used a C–R symmetry *f=*1, whereas in **b** we used symmetry *f=*0.8. To help minimize the variation between simulations for each value of mean interaction strength (*r*) we generated 100 random network configurations and then 100 samples or the community matrix and reported the mean real part of the dominant eigenvalue as the stability for that configuration. The results show that increasing the realism of the network topology has no effect on the stability of the C–R food web unless Niche like topological network structure is used.

Using the topological models mentioned above in the random community matrix context where we first look at the symmetrical case (*f=*1) in Fig. 3a, we see a surprising pattern where the extra biological information encoded in the Cascade and Generalized Cascade give not stability change from the purely Random. Only once we used the Niche model we got a difference, though it is worth noting that the qualitative shape of the stability pattern is the same (pure stabilization) but for all interaction strength values we have that the Niche model is less stable than the Random. This is a visualization of the unintuitive result found in the original Allesina and Tang^3^ paper. The puzzle of why the natural biological structure appears destabilizing becomes more interesting when we look at the non-symmetric case (Fig. 3b). Here we again see that the Random, Cascade and Generalized Cascade give identical stability results, whereas the Niche gives a qualitatively similar (checkmark) pattern it differs numerically. In the non-symmetric case, we see that for low mean interaction strength the Random-like models are more stable, analogous to the symmetric case, but once mean interaction strength gets large enough suddenly this pattern switches and the more realistic Niche model is more stable. The tradeoff of the stability dominance between these classes of models is intriguing and speaks to some underlying structure that biological networks possess that buffers against strong mean interaction strengths at the expense of weaker interactions. Moreover, the only way that this stability tradeoff can be seen is when we consider the non-symmetric case instead of the degenerate symmetric case.

## Discussion

In summary, we have shown that food web models of consumptive interactions, from single species up to assemblages of hundreds of interacting species, tend to generate a consistent stability pattern whereby increasing mean interaction strength generates a stability ‘checkmark' (Fig. 2). The only exception we found to this stability pattern occurs for the point of exact symmetry (*f=*1). This symmetry occurs where, on average, the reciprocal effects of consumers on resources, and resources on consumers, are the same in the community matrix. It is important to note that all other instances of *f* (either *f*<1 or *f*>1) produce the stability checkmark.

Thus, our result suggests that the likelihood of the real world producing pure stabilization with increasing interaction strength is extremely unlikely. Furthermore, once *f* surpasses 1 (that is, *f*>1 or very top heavy C:R ratios), we find that this only exacerbates the checkmark by shortening the region of stabilization before rapidly destabilizing the system (Supplementary Note 7). Again, this result resonates with much of C–R theory where top–heavy interactions tend to produce less stable dynamics^22^,^23^. While empirical patterns generally suggest that C:R biomass ratios are less than 1 (refs 14, 15), some aquatic systems have been argued to be inverted^14^. Nonetheless, our results suggest that both (*f*<1 and *f*>1) are expected to be destabilized by strong interactions; further, we expect the more top-heavy aquatic webs (potentially *f*>1) to be more greatly destabilized. This is necessarily true unless the real world, for some unknown reason, is able to constrain webs to exactly *f=*1 case.

Furthermore, our analysis shows that growth, interaction strength and lags interact in a general way to produce this same consistent stability result across the ecological hierarchy. Increasing growth, flux or interaction strength through a population or interacting assemblage, first drives a stabilizing phase before entering a subsequent destabilizing phase (stability checkmark). In low-diversity dynamic models, we have shown that the destabilizing phase corresponds to increased population dynamic overshoot (Supplementary Notes 1–4) and eventually cycles or chaos in many models. In a sense, high energy or growth drives the dynamics to express the inherent biological lags (population lags or C–R lags) and, once expressed, further increases and excites these lags to greater instability.

A recent synthetic data result that compares aquatic ecosystem dynamics to terrestrial ecosystem dynamics allows a preliminary examination of our whole-web results. Specifically, aquatic ecosystems, which have smaller body size and more palatable resources than terrestrial ecosystems, tend to have larger interaction strengths than terrestrial ecosystems^24^,^25^,^26^. Consistent with the results presented here, strongly interacting aquatic ecosystems also appear to empirically have more top heavy webs than terrestrial ecosystems (that is, aquatic webs have more biomass in the higher trophic levels^22^). This higher trophic level accumulation of biomass is symptomatic of high energy flow or growth throughout the aquatic food web^22^. Finally, aquatic ecosystems show significantly more population dynamic variance than terrestrial ecosystems^22^, suggesting that the strong interactions in aquatic ecosystems, in fact, decrease stability. Thus, we argue that both theoretical and empirical evidence suggest strong interactions are indeed destabilizing.

The corollary of the result that strong interactions tend to be destabilizing is that weak interactions are, indeed, fundamental to the maintenance of diversity. Recent analyses of empirical complexity–stability relationships have found little relationship between stability and classical metrics of stability^27^; however, intriguingly one of the major arguments for this discrepancy relied on the empirical finding that the interaction strength distribution became increasingly skewed towards weak interactions as diversity increased. Thus, our result, which resonates with consumptive food web theory across scales, is also consistent with recent empirical whole food web stability results.

## Methods

Numerical simulations were carried out using Mathematica and the Julia language^28^, both calling standard numerical procedures for linear algebra found in LAPACK^29^. Random community matrices were generated by filling a double precision *S* × *S* array, where *S* is the number of species in the simulation, with either zero or non-zero values according to the specified network model (Erdos-Reyni^4^, Cascade^30^, Generalized Cascade^20^ or Niche^21^). Once the non-zero locations of the array were found they were filled by simulating a pair of independent random values from a given distribution (Uniform, Normal, or Gamma). For distributions that could take on negative values, the absolute value of the samples was taken so that the exact sign-structure could be enforced. For each pair of random values one was forced to be negative, and the other, positive valued, element was scaled by the desired symmetry value *f*. These scaled and signed pair of random, independent, values were then placed in the numerical array, with one value occurring at the (*i*, *j*) location and the other occurring in the (*j*, *i*) location, thereby forming a pair symmetric around the diagonal of the square matrix. Once all non-zero values were filled in this manner then eigenvalues were calculated, stability was determined by the real part of dominant eigenvalue.

## Additional Information

**How to cite this article:** Gellner, G. and McCann, K. S. Consistent role of weak and strong interactions in high- and low-diversity trophic food webs. *Nat. Commun.* 7:11180 doi: 10.1038/ncomms11180 (2016).

## Supplementary Material

Supplementary Information Supplementary Figures 1-18, Supplementary Tables 1-2, Supplementary Notes 1-7 and Supplementary References.
