# Supplementary material for: Consistent role of weak and strong interactions in high- and low-diversity trophic food webs
Source: Nat Commun. 2016 Apr 12;7:11180. doi: 10.1038/ncomms11180 (PMC4832055; doi:10.1038/ncomms11180)
Supplement: Supplementary Information — Supplementary Figures 1-18, Supplementary Tables 1-2, Supplementary Notes 1-7 and Supplementary References. [file ncomms11180-s1.pdf]

Supplementary Figure 1

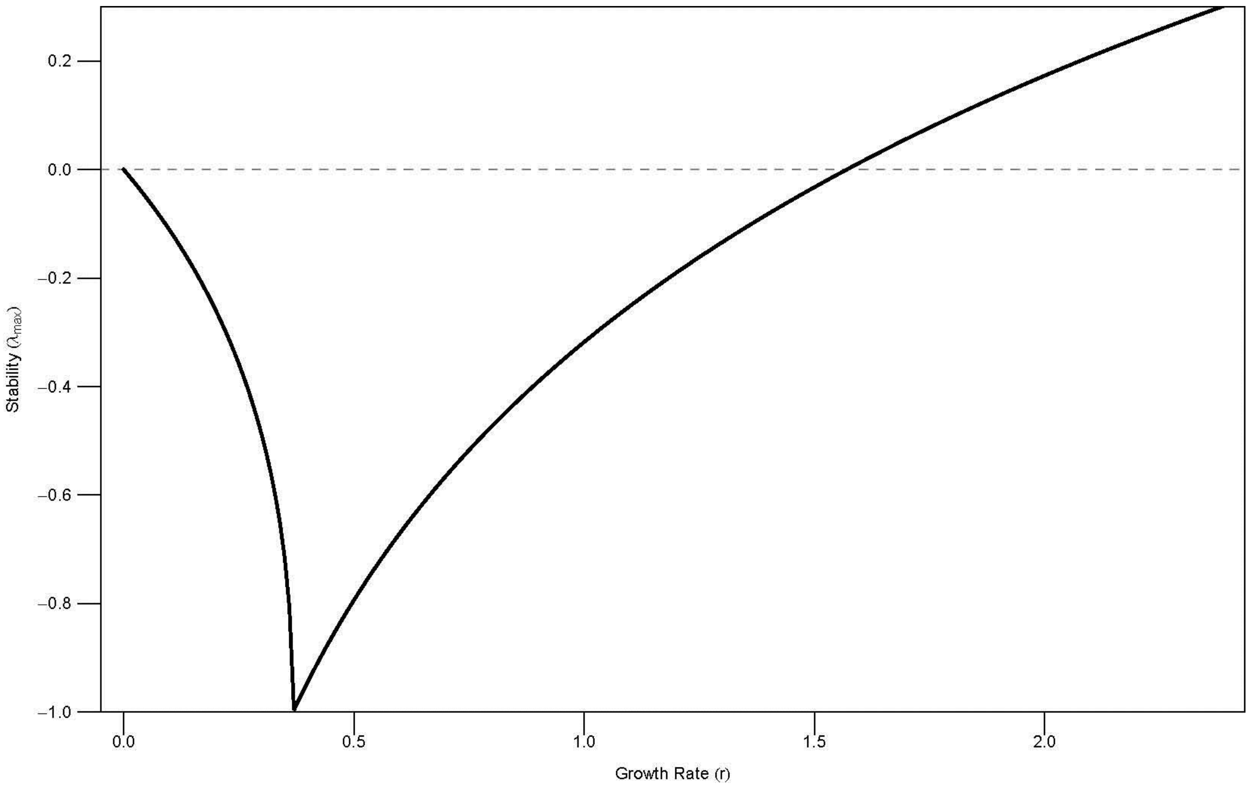

Supplementary Figure 1: The stability response to increasing r for the lagged logistic model with parameters K=1.0, tau=1. Stability is measured by the real part of the dominant eigenvalue.

Supplementary Figure 2

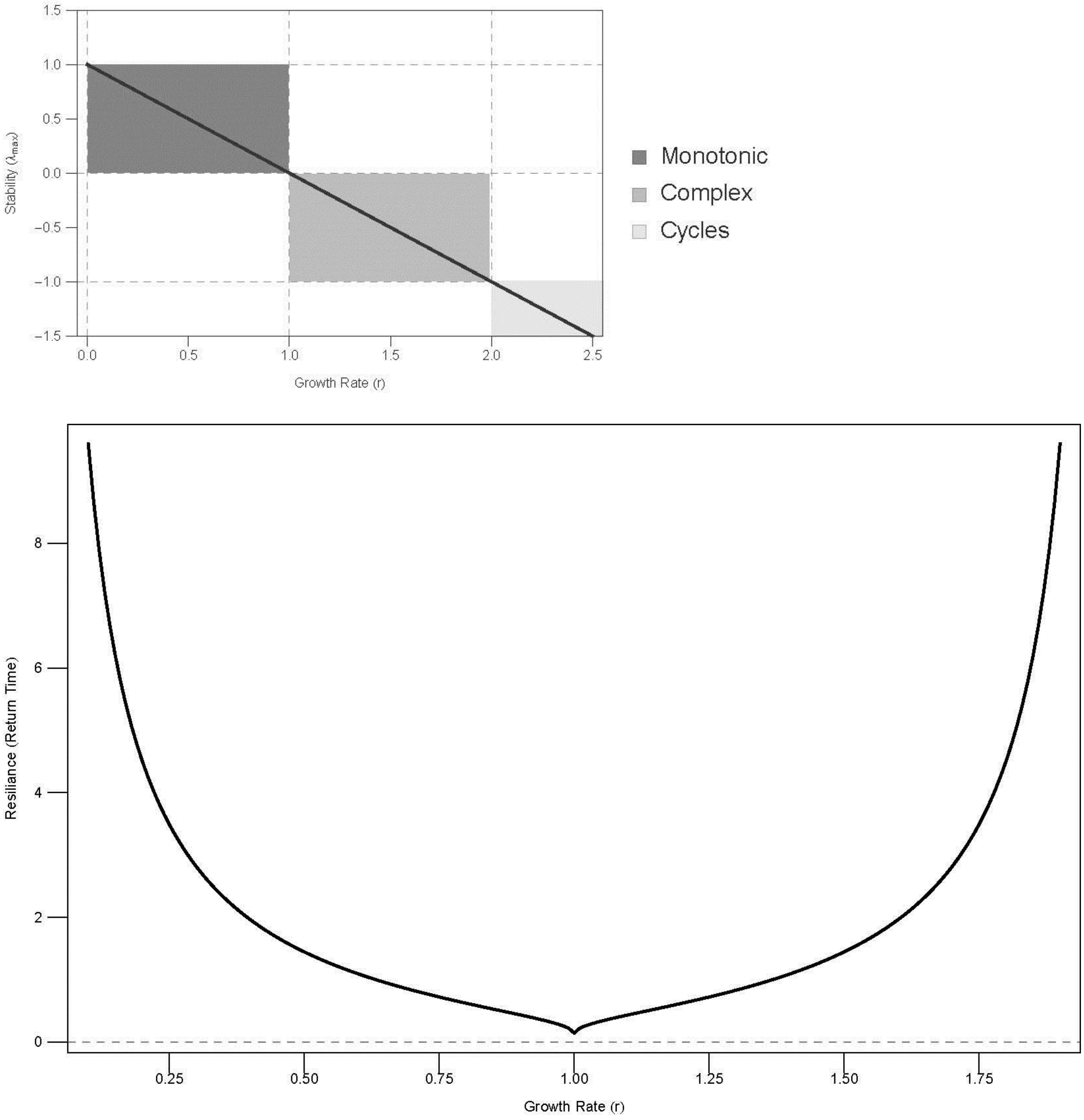

Supplementary Figure 2: The stability of the Ricker logistic equation with parameters k=1.0. In panel (a) we show stability as measured by the real part of the dominant eigenvalue. As the model is discrete the interpretation of stability is slightly different than for continuous models. Specifically, stability occurs when the real part of the dominant eigenvalue is between -1 and 1, this region is labeled by the monotonic and complex region. The region labeled cycles begins once the real part of the dominant eigenvalue is less than -1. To make the stability relationship between the discrete and continuous case more explicit we also show the resilience of this model for changes in r in panel (b). This represents the identical information for stability but the y-axis now has an analogous scale as for the continuous case where decreasing along the y-axis represents increasing stability. On this scale we can clearly see the checkmark stability pattern

Supplementary Figure 3

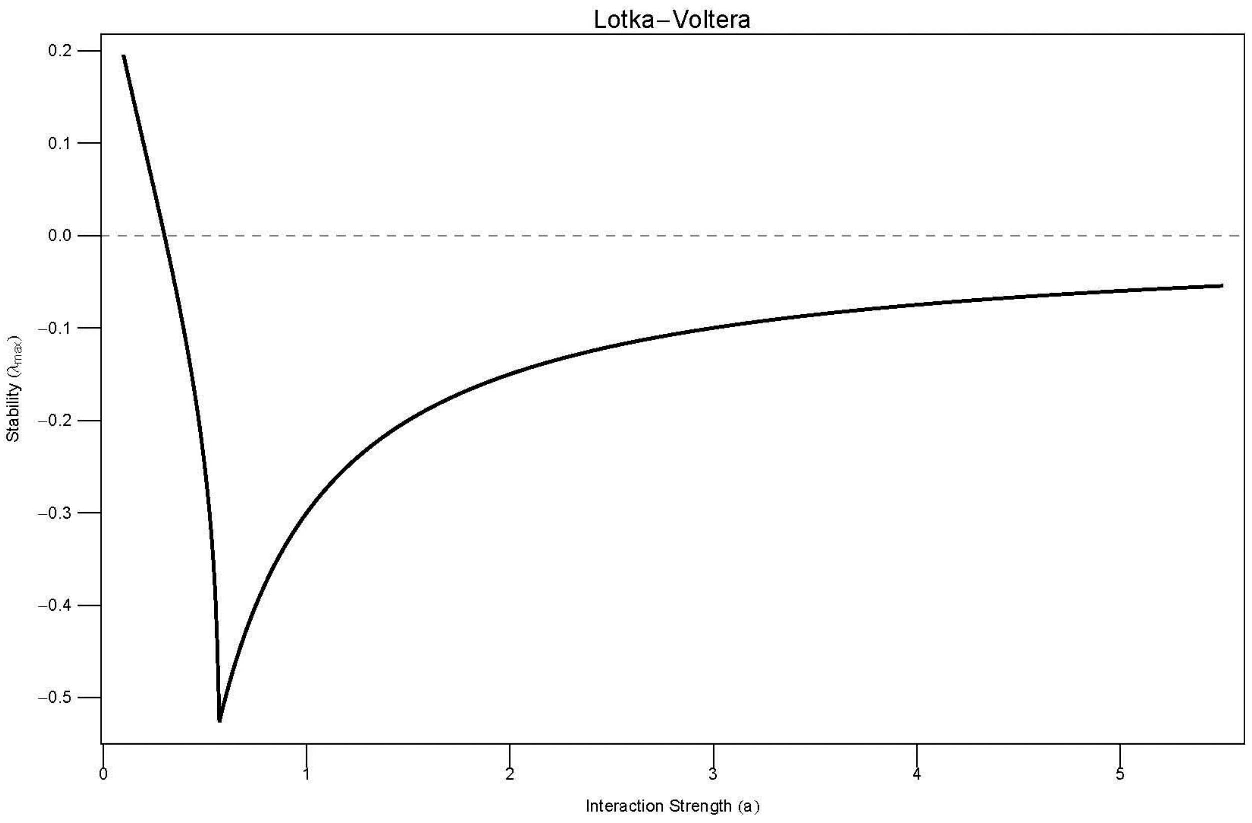

Supplementary Figure 3: The stability response to increasing consumer attack rate a, a measure of the interaction strength of the consumer-resource interaction. Here we show a Lotka-Voltera (type I) functional response with parameters r=2.0, K=1.0, e=1.0, m=0.3. Stability is measured as the real part of the dominant eigenvalue. Clearly we see the checkmark stability tradeoff as interaction strength (a) is increased. Notice that the model is never fully destabilized with sustained oscillations. This is expected from the type I response.

Supplementary Figure 4

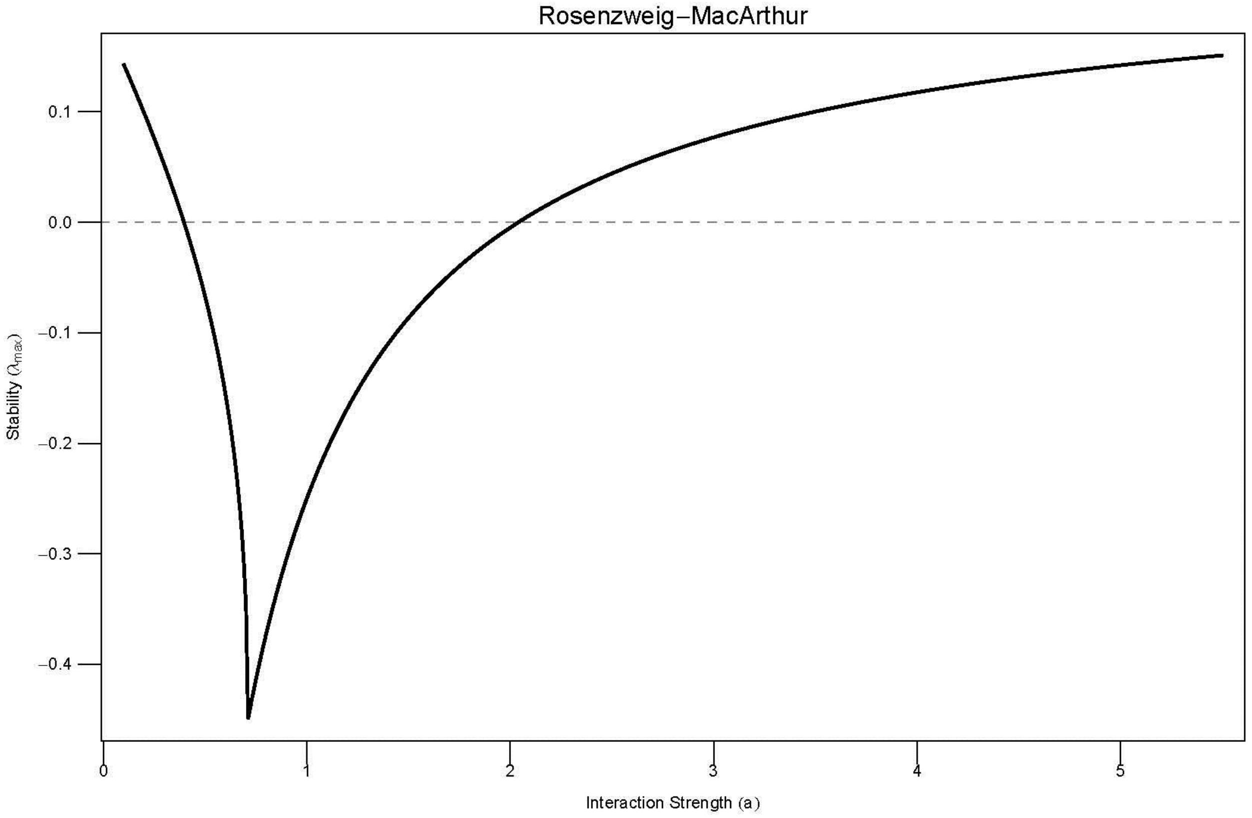

Supplementary Figure 4: The stability response to increasing consumer attack rate a, a measure of the interaction strength of the consumer-resource interaction. Here we show a Rosenzweig-MacArthur (type II) functional response with parameters r=2.0, K=1.0, e=1.0, m=0.3, h=0.8. Stability is measured as the real part of the dominant eigenvalue. Clearly we see the checkmark stability tradeoff as interaction strength (a) is increased. In contrast to the type I response the type II can lose its stable equilibrium and exhibit sustained oscillations shown here when the dominant eigenvalue becomes positive a little past a=2.0.

Supplementary Figure 5

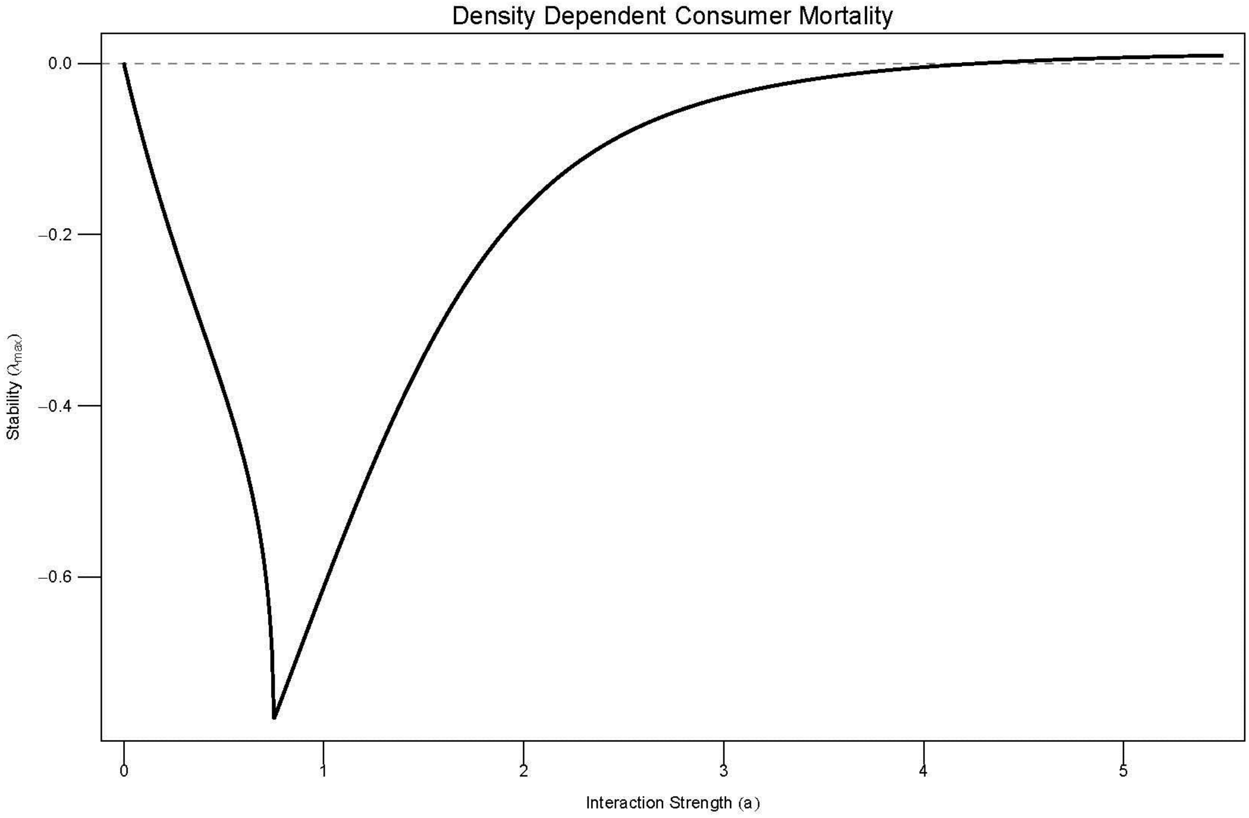

Supplementary Figure 5: The stability response to increasing consumer attack rate a, a measure of the interaction strength of the consumer-resource interaction. Here we show a type II model with quadratic density dependent consumer mortality with parameters r=2.0, K=1.0, e=1.0, m=0.3, h=0.8. Stability is measured as the real part of the dominant eigenvalue.

Supplementary Figure 6

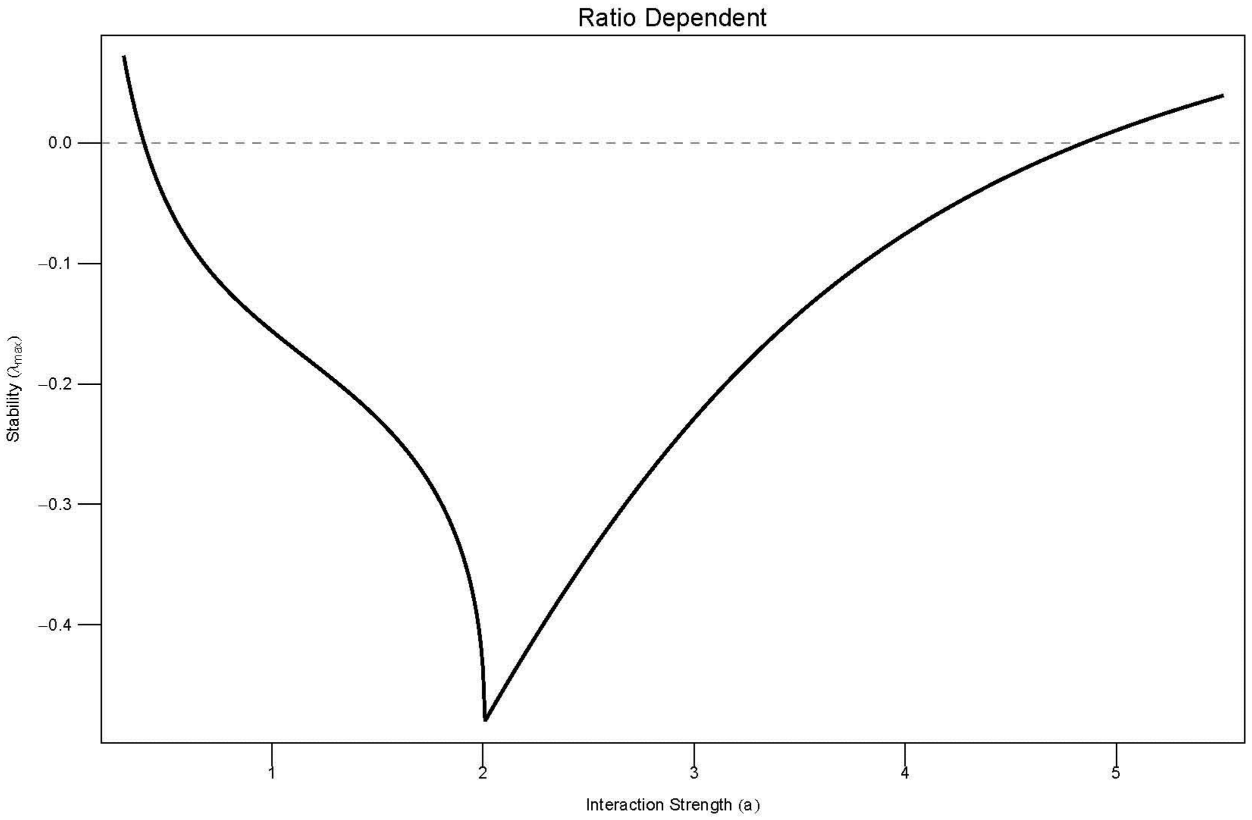

Supplementary Figure 6: The stability response to increasing consumer attack rate a, a measure of the interaction strength of the consumer-resource interaction. Here we show a type II model with quadratic density dependent consumer mortality with parameters r=2.0, K=1.0, e=1.0, m=0.3, h=0.8, gamma=1.0. Stability is measured as the real part of the dominant eigenvalue.

Supplementary Figure 7

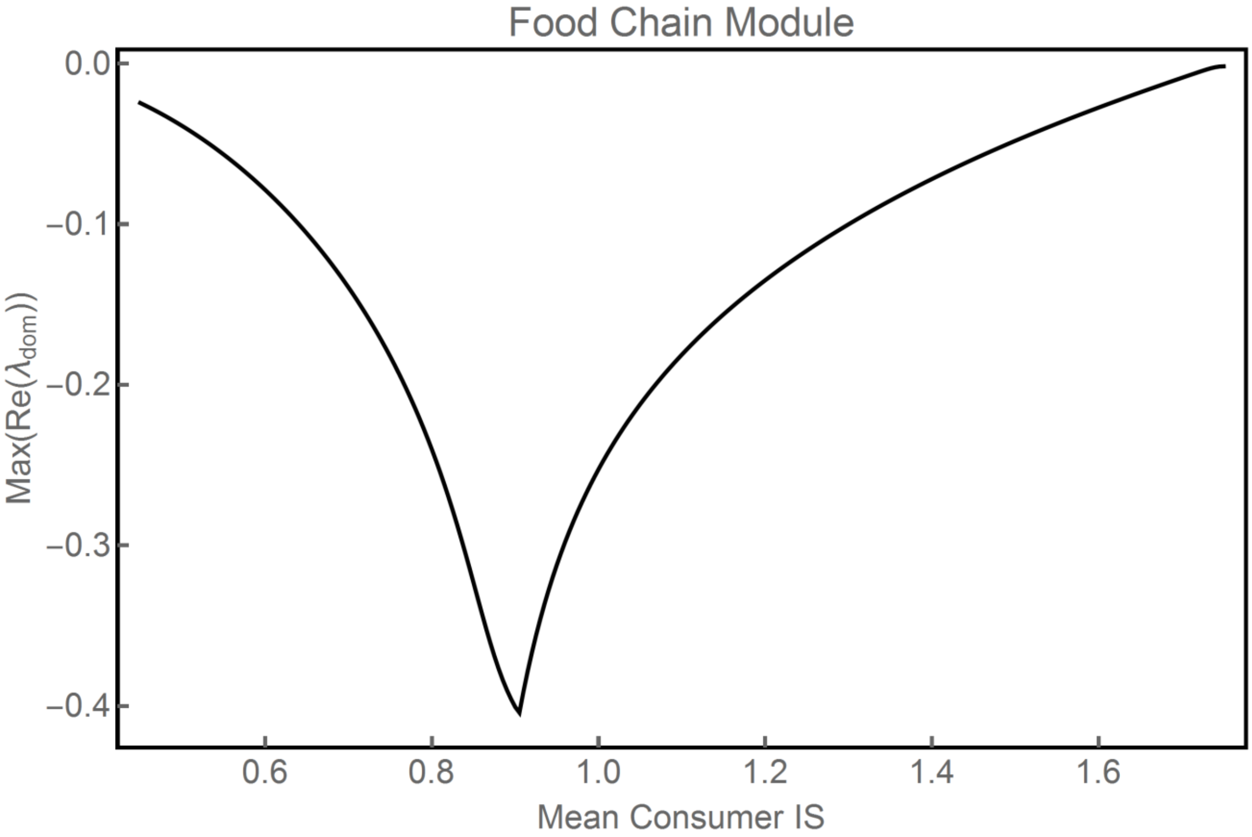

Supplementary Figure 7: Adding a top predator to the consumer-resource models gives a qualitatively similar pattern, the stability checkmark, for increasing the mean interaction strength of the top predator and the consumer on the resource. Model parameters: r=2.0, K=1.0, eRC=1.0, eCP=1.0, mC=0.3, mP=0.3, aCP=0.5, with aCR changing from 0.4 to 0.3 in steps of 0.01.

Supplementary Figure 8

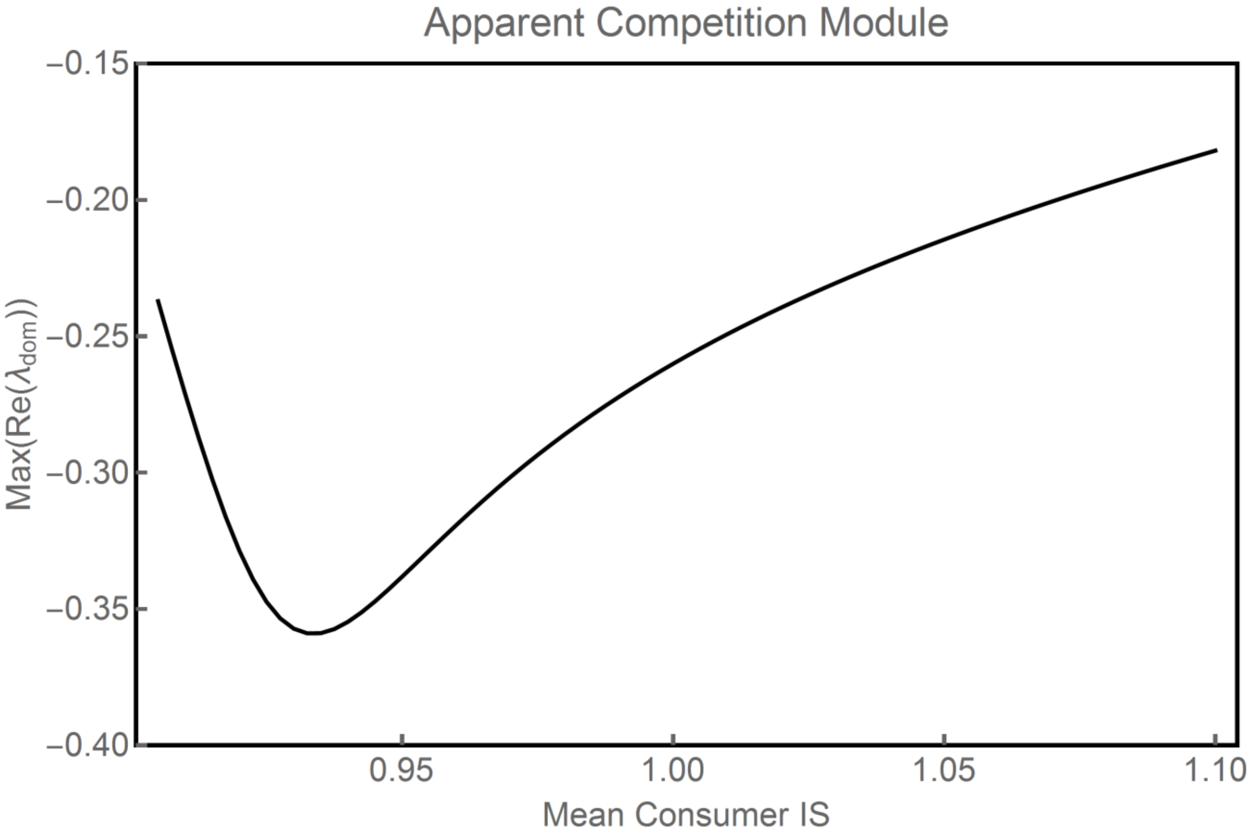

Supplementary Figure 8: The stability response of the three species apparent competition module gives the stability checkmark as the mean interaction strength of the top, generalist, consumer is increased. In this case we increase the interaction on one of the two resources (a1), but qualitatively similar patterns occur for increasing both. Model parameters: r1=0.5, r2=0.4, K1=10.0, K2=10.0, e1=1.0, e2=1.0, m=1.0, a2=1.7, with a1 changing from 0.11 to 0.5 in steps of 0.005.

Supplementary Figure 9

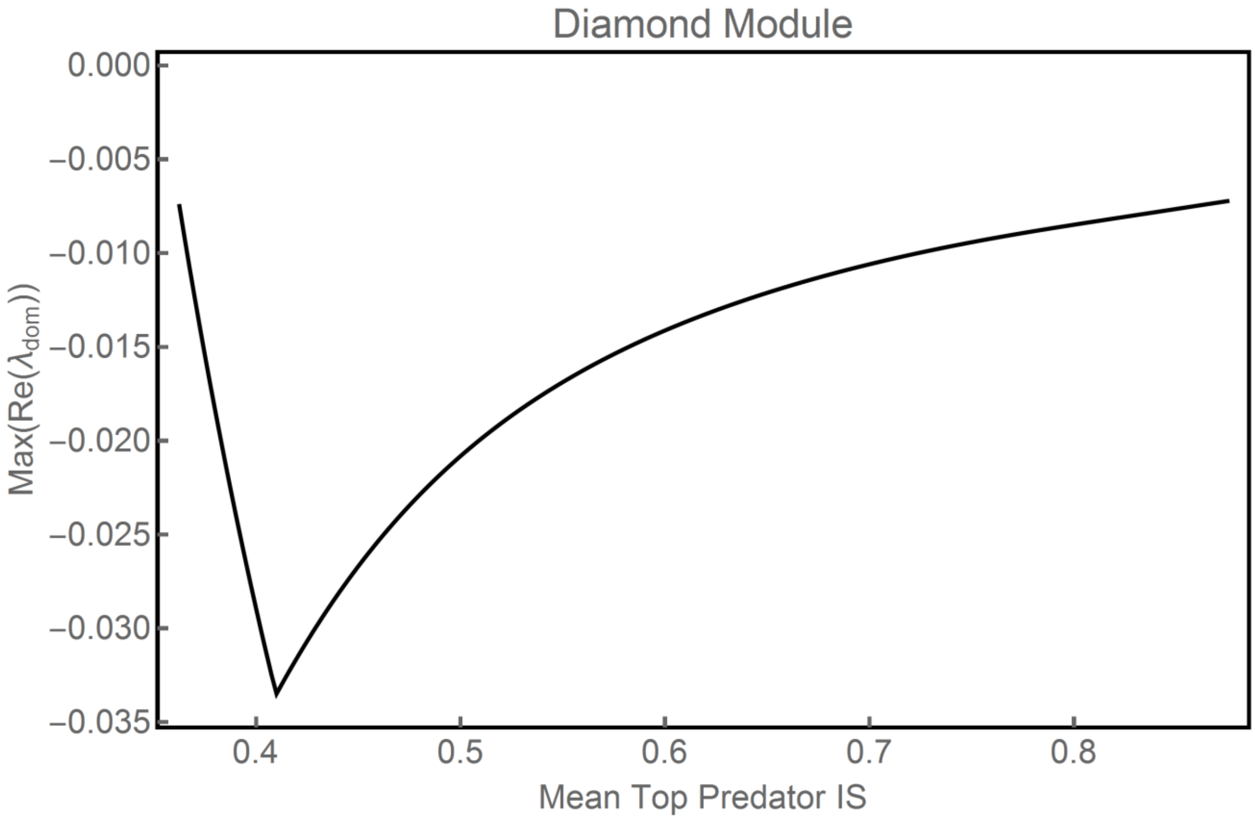

Supplementary Figure 9: Stability response for increasing the interaction strength of the top predator (aC1P) of the four species "diamond" module. We again see the familiar checkmark stability pattern. Model parameters: r=2.0, K=1.0, e=1.0, aRC1=0.5, aRC2=0.4, mC1=0.3, mC2=0.25, mP=0.3, aC2P=0.1, with aC1P changing from 0.45 to 2.5 in steps of 0.01.

Supplementary Figure 10

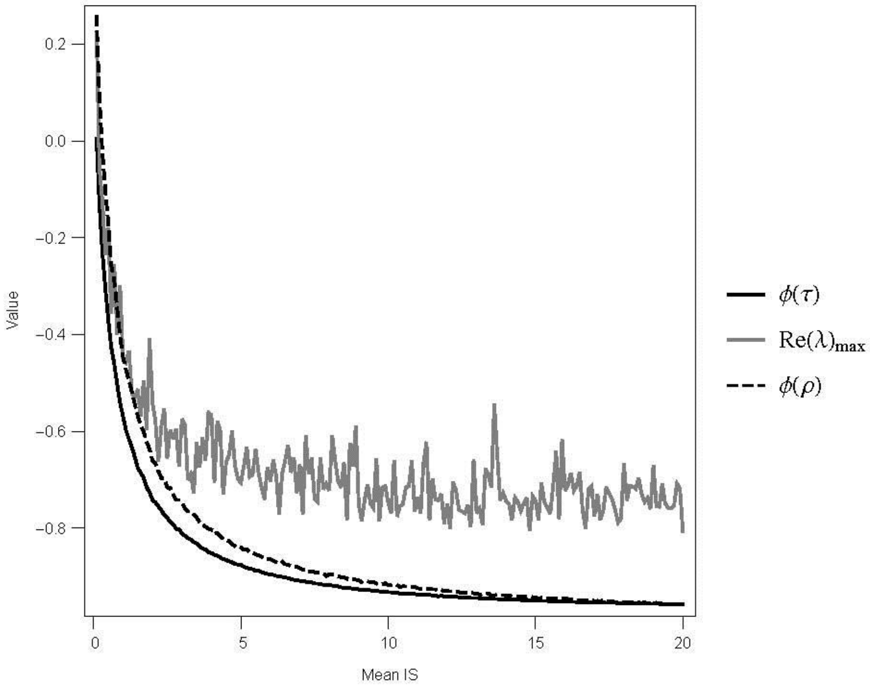

Supplementary Figure 10: Test of the different forms for the damping term (x) in the stability metric $\phi(x) = \sigma\sqrt{SC}(1+x)$. We try the Allesina and Tang estimate τ as well as the true correlation ρ, and compare these to the true stability $Re(\lambda)_{\max}$. Over this range of mean interaction strength we see a close qualitative match between all three metrics. With the derived estimates having a bias to greater stability. Model parameters are S=250, C=0.25, f=1, with the distribution of IS being drawn from a Uniform(r, r+1), with mean IS equal to (2r + 1)/2 and constant variance 1/12.

Supplementary Figure 11

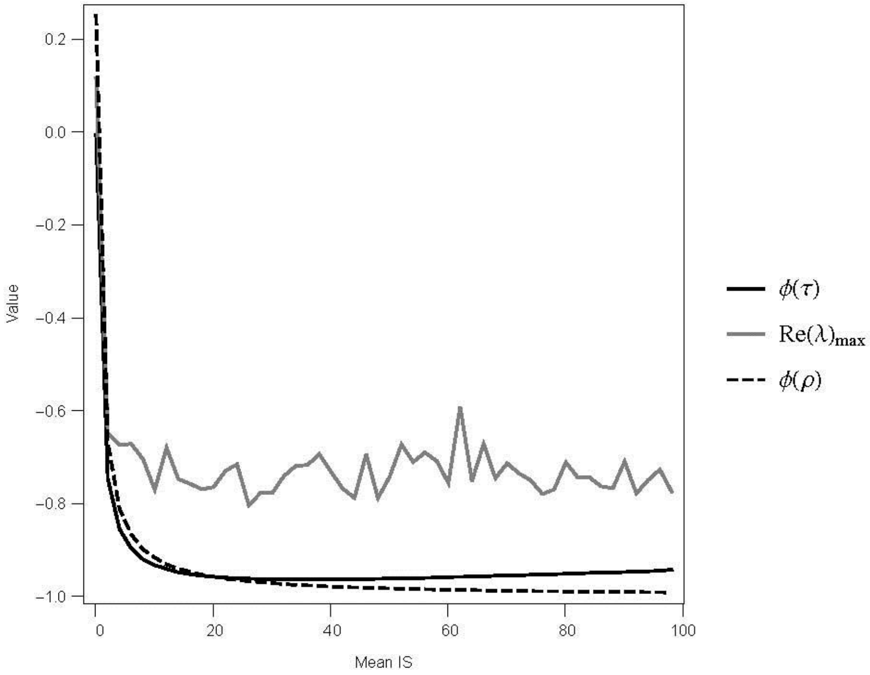

Supplementary Figure 11: Test of the different forms for the damping term (x) in the stability metric $\phi(x) = \sigma\sqrt{SC}(1+x)$. We try the Allesina and Tang estimate τ as well as the true correlation ρ, and compare these to the true stability $Re(\lambda)_m ax$. Over a larger range of mean IS we begin to see the Allesina and Tang τ beginning to show decreasing stability, no longer being close to the actual model correlation. Model parameters are S=250, C=0.25, f=1, with the distribution of IS being drawn from a Uniform(r, r+1), with mean IS equal to (2r + 1)/2 and constant variance 1/12.

Supplementary Figure 12

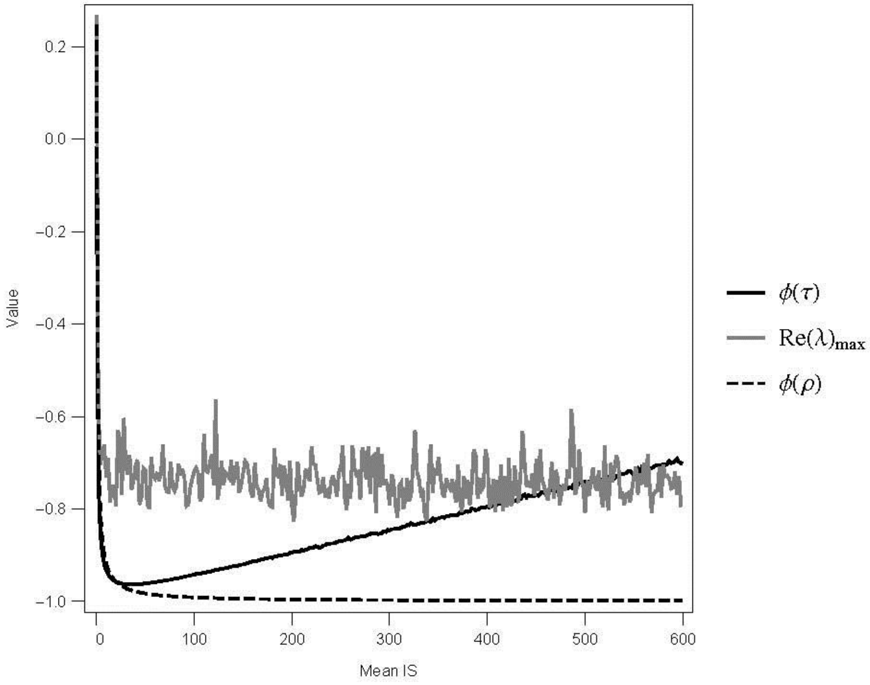

Supplementary Figure 12: Test of the different forms for the damping term (x) in the stability metric $\phi(x) = \sigma\sqrt{SC}(1+x)$. We try the Allesina and Tang estimate τ as well as the true correlation ρ, and compare these to the true stability $Re(\lambda)_{\max}$. At extreme levels of mean IS the Allesina and Tang estimate τ deviates strongly from the model correlation, suggesting a stability checkmark when in fact there is none. Model parameters are S=250, C=0.25, f=1, with the distribution of IS being drawn from a Uniform(r, r+1), with mean IS equal to (2r + 1)/2 and constant variance 1/12.

Supplementary Figure 13

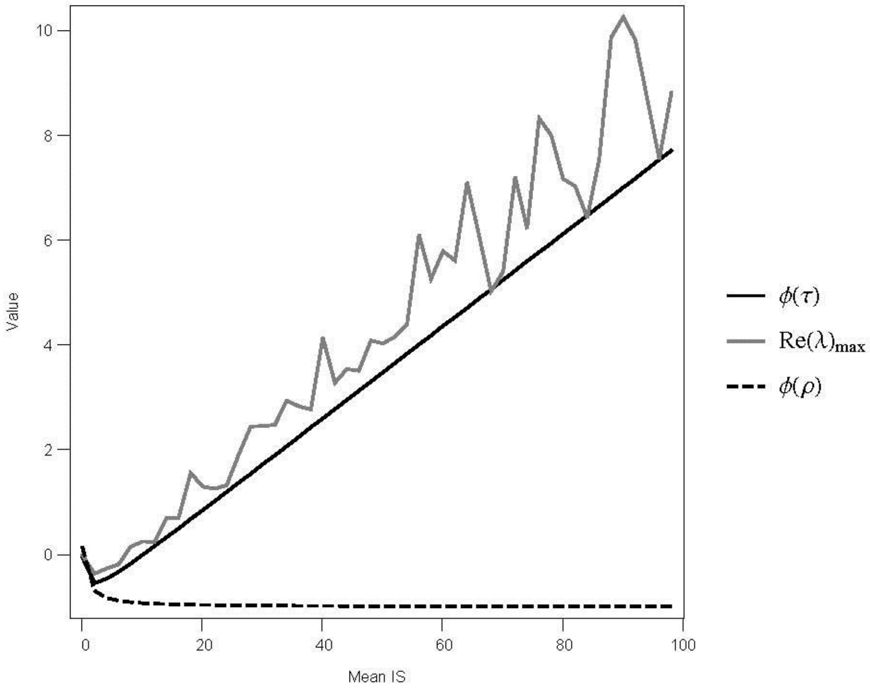

Supplementary Figure 13: Test of the different forms for the damping term (x) in the stability metric $\phi(x) = \sigma\sqrt{SC}(1+x)$. We try the Allesina and Tang estimate τ as well as the true correlation ρ, and compare these to the true stability $Re(\lambda)_{\max}$. Interestingly when $f \neq 1$ the model correlation no longer gives a good description of the stability, whereas Allesina and Tangs τ gives a close approximation. Model parameters are S=250, C=0.25, with the distribution of IS being drawn from a Uniform(r, r+1), with mean IS equal to (2r + 1)/2 and constant variance 1/12.

Supplementary Figure 14

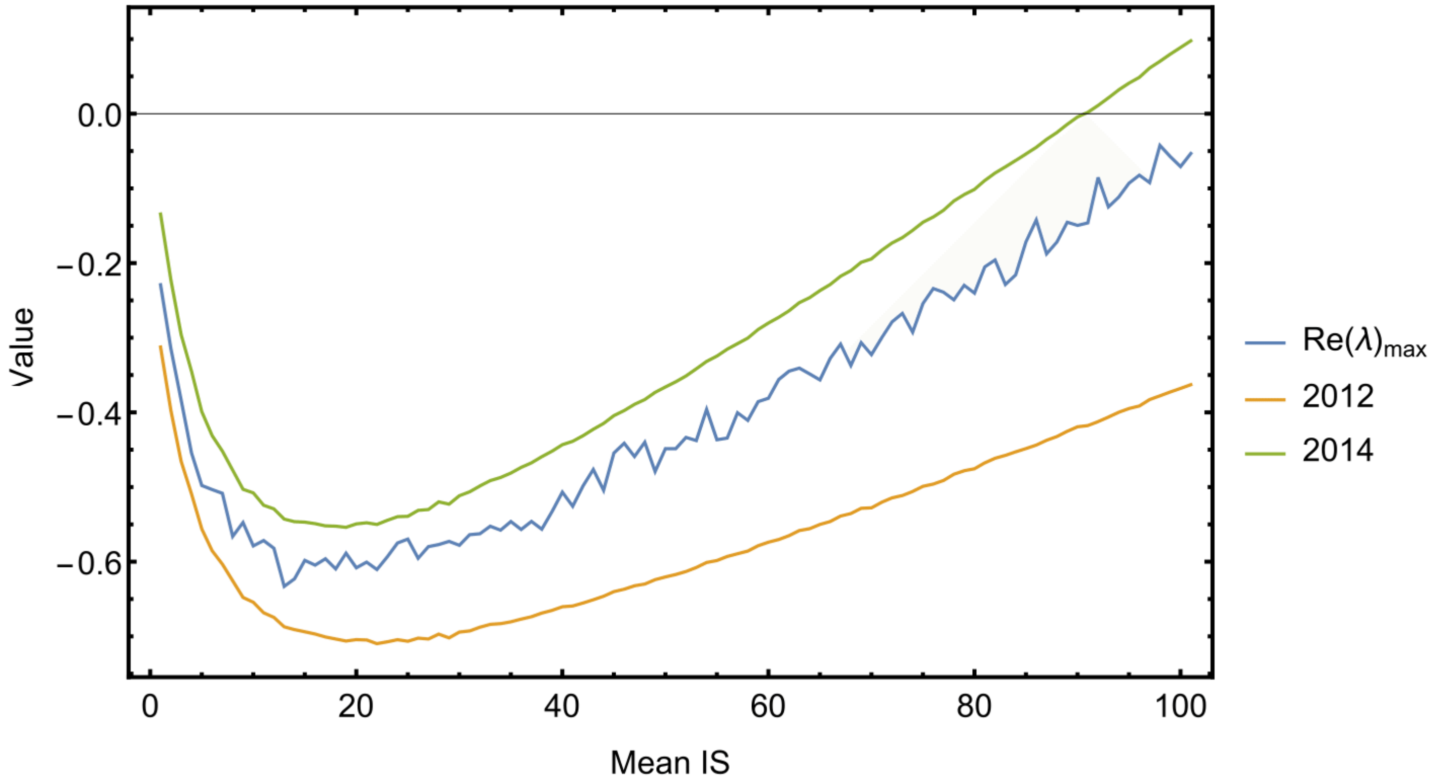

Supplementary Figure 14: Comparing the different forms of the metric used to estimate the dominant eigenvalue $Re(\lambda)_{max}$. He we see older estimate that doesn't consider asymmetric community matrices (2012) versus the more recent estimate (2014) that gives another form. In this example we see that the two forms act as upper and lower bounds for the estimate each giving a qualitatively similar pattern. It should be noted that for larger or smaller values of S and C the real eigenvalue bound can be closer to either estimate, and even be outside of the range. Model parameters: S=100, C=0.25, Predator-Prey pairs drawn from Uniform(r, r+1.0), where r increases from 0 to 10 in steps of 0.1.

Supplementary Figure 15

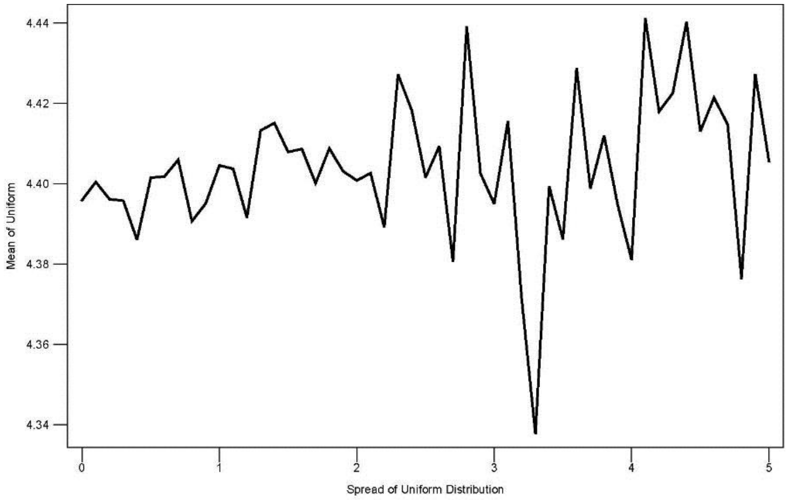

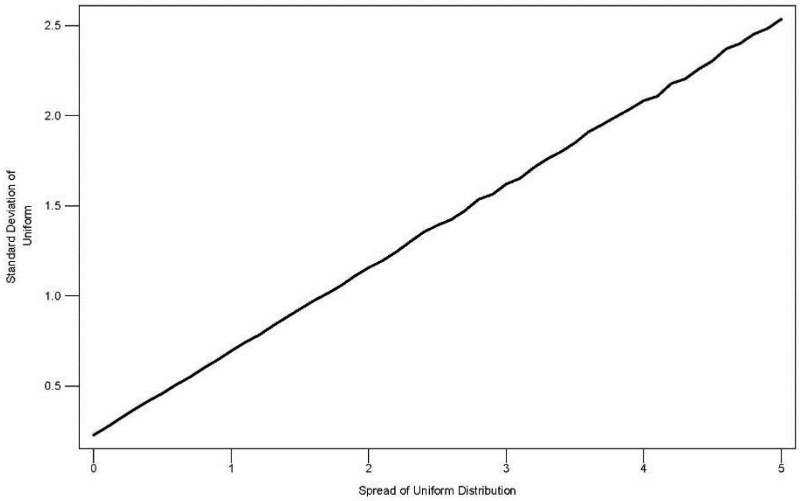

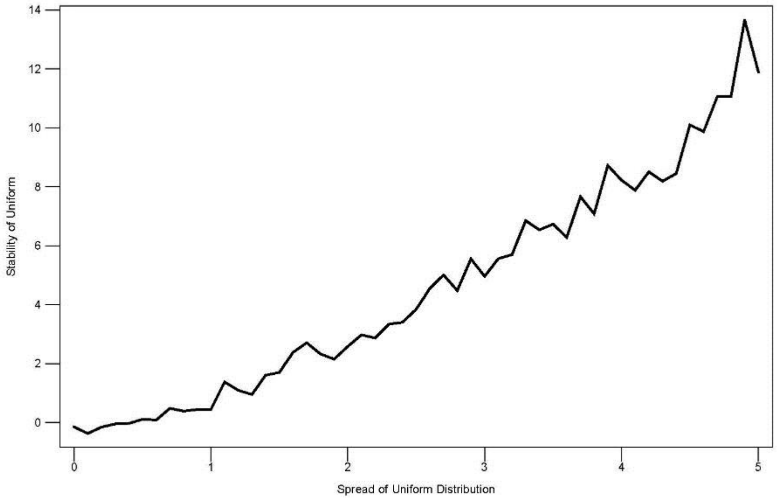

Supplementary Figure 15: The relationship between stability and increasing half distribution variance increasing while half distribution mean is kept equal. The random community matrix with parameters S=250, C=0.25, f=0.8, and the interactions strength distribution is Uniform(a-r, b+r) with a=5, b=6 and r ranging from 0 to 5. Panel (a) shows that our modeling setup is correct with the mean of the half distribution not being changed. Panel (b) shows that we get an increase in the standard deviation of the half distribution. Panel (c) shows that when this pure variance increase occurs that we get pure destabilization.

Supplementary Figure 16

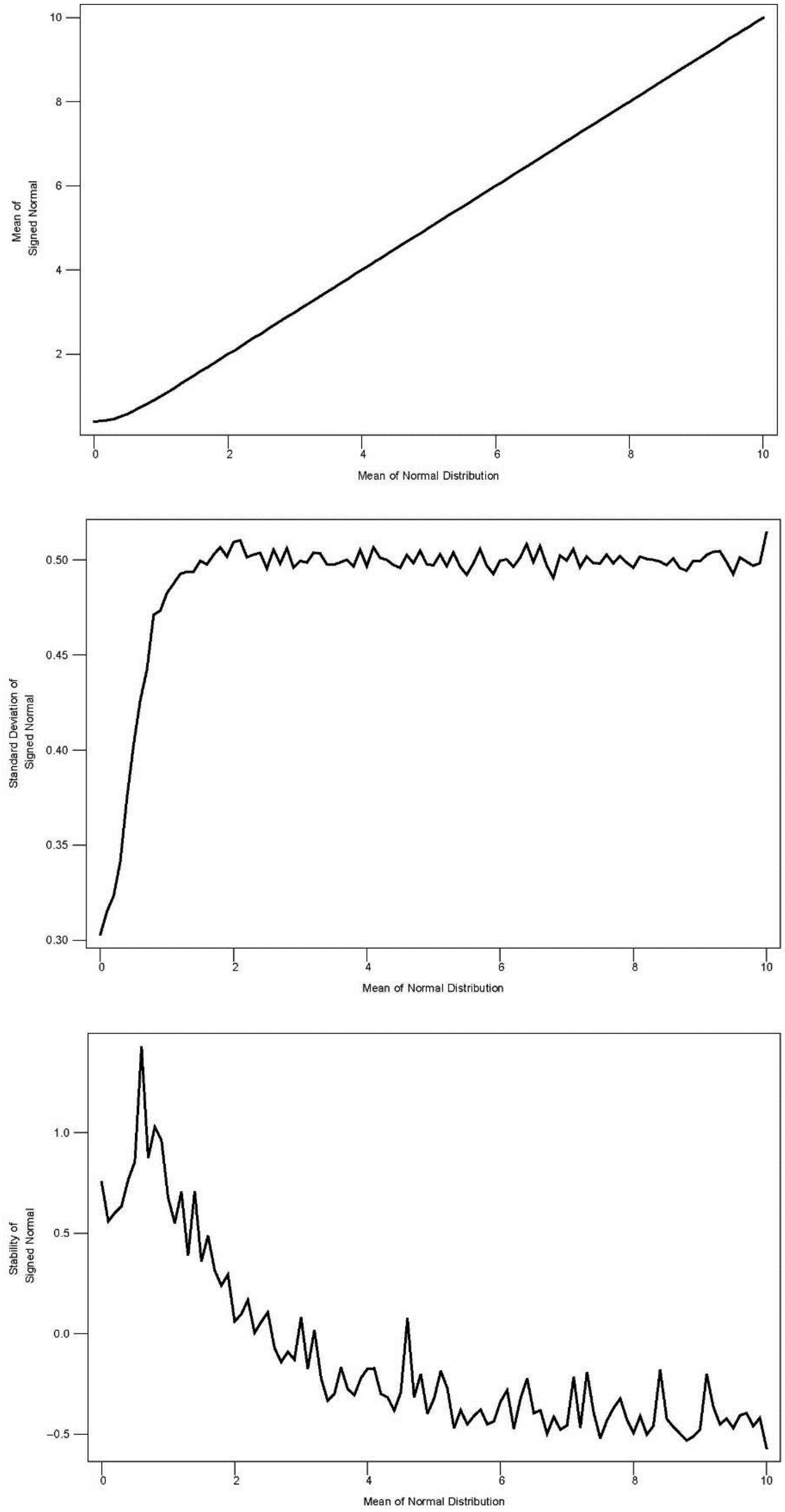

Supplementary Figure 16: The relationship between stability and increasing half distribution variance increasing while half distribution mean is kept equal. The random community matrix with parameters S=250, C=0.25, f=1.0, and the interactions strength distribution is Abs(Normal(r, 0.5)) with r ranging from 0 to 10 in increments of 0.1. Panel (a) shows that the mean increases almost linearly. Panel (b) shows that we get an initially sharp increase in the standard deviation of the half distribution, caused by the distortion caused by taking the absolute value of the normal. Once the mean of the underlying Normal distribution (r) gets large enough this distortion is minimized and the standard deviation stabilizes. Panel (c) shows the stability response for this mixed effect of mean and variance changes. At first the dramatic change in the standard deviation outweighs the stabilizing effect of the weak mean effect causing an initial destabilization for weak mean interaction strength. Once the variance stops changing we see the symmetric pure stabilizing response shown in the main text.

Supplementary Figure 17

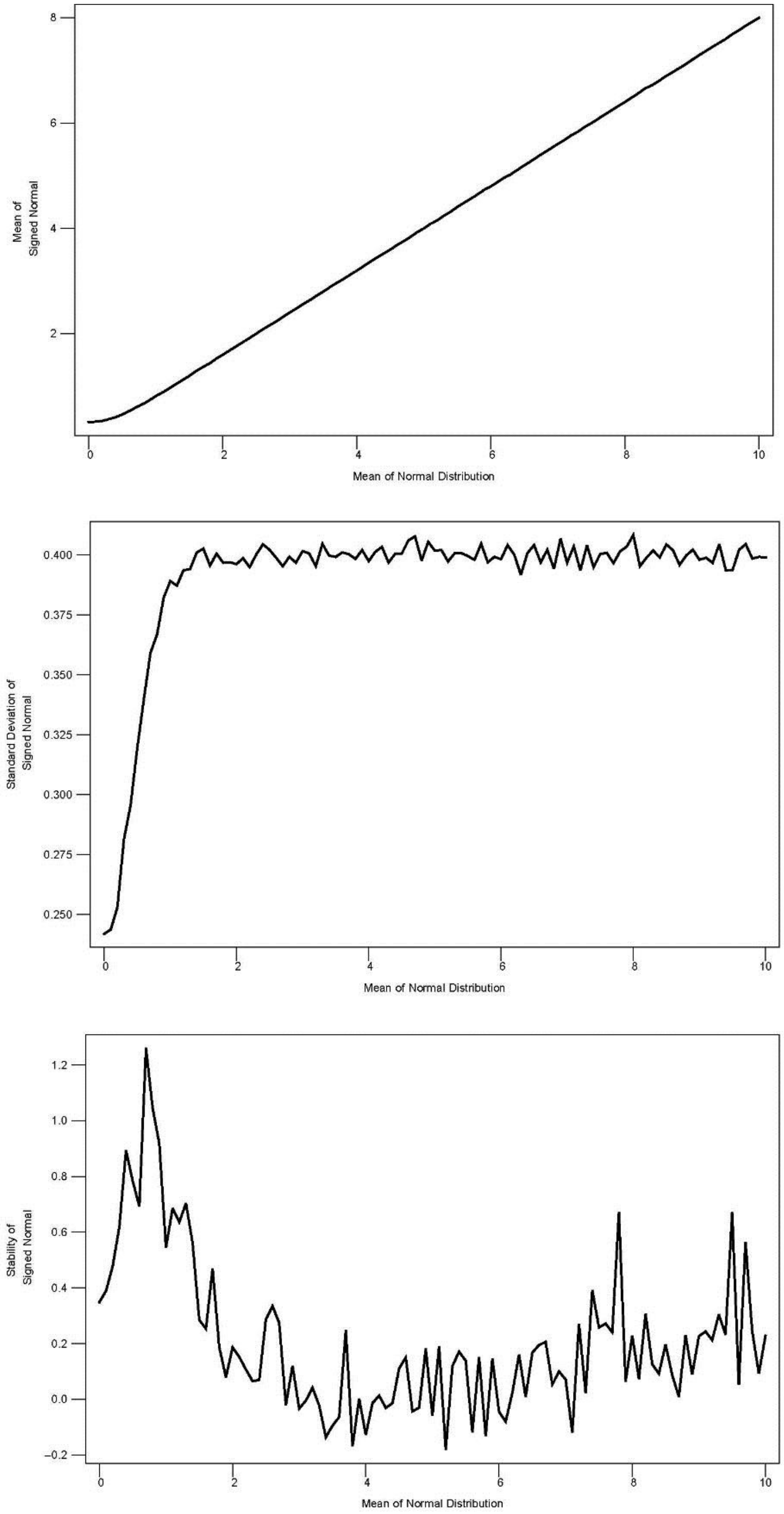

Supplementary Figure 17: The relationship between stability and increasing half distribution variance increasing while half distribution mean is kept equal. The random community matrix with parameters S=250, C=0.25, f=1.0, and the interactions strength distribution is Abs(Normal(r, 0.5)) with r ranging from 0 to 10 in increments of 0.1. Panel (a) shows that the mean increases almost linearly. Panel (b) shows that we get an initially sharp increase in the standard deviation of the half distribution, caused by the distortion caused by taking the absolute value of the normal. Once the mean of the underlying Normal distribution (r) gets large enough this distortion is minimized and the standard deviation stabilizes. Panel (c) shows the stability response for this mixed effect of mean and variance changes. At first the dramatic change in the standard deviation out outweighs the stabilizing effect of the weak mean effect causing an initial destabilization for weak mean interaction strength. Once the variance stops changing we see the expected effect of a checkmark stability pattern for the non-symmetric case.

Supplementary Figure 18

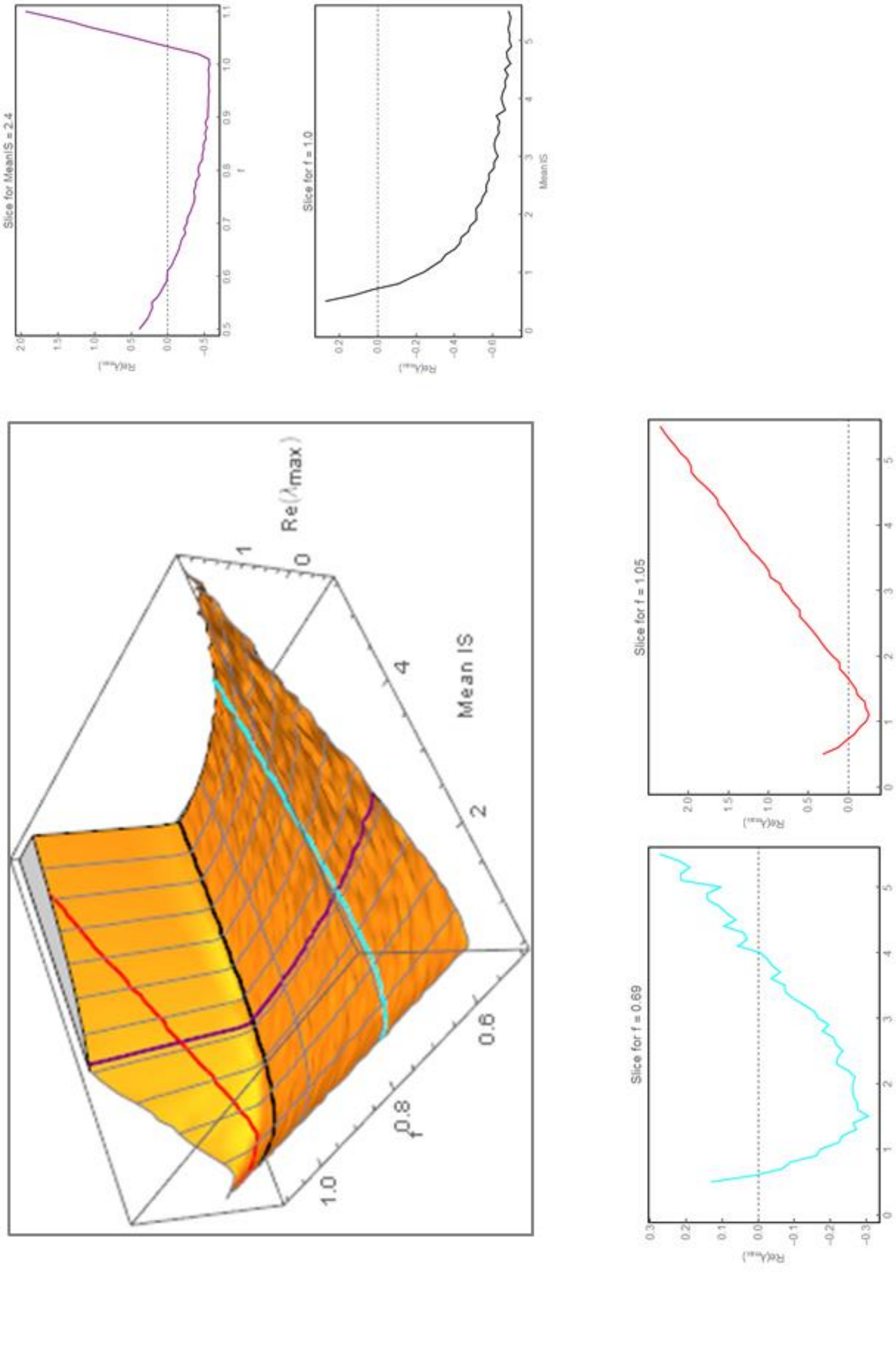

Supplementary Figure 18: Changing both Mean IS and f for a random community matrix with half distribution drawn from U(r, r + 1), S = 250, C = 0.25, f in [0.5, 1.1], r in [0, 5]. Stability at each parameter combination was resampled 10 times for each instance of a random network and the mean real part of the dominant eigenvalue was used. For each of these mean stability points 15 different network topologies where generated and each stability surface was then averaged. This removes some of the random variation around the stability points, but even single values give the same qualitative pattern. Higher resampling/network gives smoother figures, but as can be seen even for these low values the pattern is quite smooth. We have highlighted representative slices along each axis, where corresponding colors in the 3D surface correspond to the 2D slices.

Supplementary Table 1

Module	Equations	Parameters
Omnivory	$\frac{dR}{dt} = rR(1 - R/K) - a_{RC}\,\mathrm{RC} - a_{RP}RP$<br>$\frac{dC}{dt} = e_{RC}a_{RC}\,\mathrm{RC} - \mathrm{a}_{CP}\,CP - m_C$<br>$\frac{dP}{dt} = e_{RP}a_{RP}\,\mathrm{RP} + e_{CP}\,\mathrm{a}_{CP}\,CP - m_P$	$r = 2.0$<br>$K = 1.0$<br>$e_{RC} = 1.0$<br>$e_{CP} = 1.0$<br>$m_C = 0.3$<br>$m_P = 0.3$<br>$a_{CP} = 0.5$<br>$a_{RC} \in [0.4, 3.0]$
Food Chain	$\frac{dR}{dt} = rR(1 - R/K) - a_{RC}\,\mathrm{RC}$<br>$\frac{dC}{dt} = e_{RC}a_{RC}\,\mathrm{RC} - \mathrm{a}_{CP}\,CP - m_C$<br>$\frac{dP}{dt} = e_{CP}\,\mathrm{a}_{CP}\,CP - m_P$	$r = 2.0$<br>$K = 1.0$<br>$e_{RC} = 1.0$<br>$e_{CP} = 1.0$<br>$m_C = 0.3$<br>$m_P = 0.3$<br>$a_{CP} = 0.5$<br>$a_{RC} \in [0.4, 3.0]$
Apparent Competition	$\frac{dR_1}{dt} = r_1R_1(1 - R_1/K_1) - a_{R_1}R_1C$<br>$\frac{dR_2}{dt} = r_2R_2(1 - R_2/K_2) - a_{R_2}R_2C$<br>$\frac{dC}{dt} = e_{R_1}a_{R_1}R_1C + e_{R_2}a_{R_2}R_2C - mC$	$r_1 = 0.5$<br>$r_2 = 0.4$<br>$K_1 = 10.0$<br>$K_2 = 10.0$<br>$e_1 = 1.0$<br>$e_2 = 1.0$<br>$m = 1.0$<br>$a_{R_2} = 1.7$<br>$a_{R_1} \in [0.11, 0.5]$

Supplementary Table 1: Food Web Modules and Parameter Values used in main text Figure 1.

Supplementary Table 2

	$\Gamma(r, \sigma\sqrt{1/(r^2+r)})$	$\Gamma(r, \sigma/\sqrt{r})$
Mean	$\sqrt{\frac{r}{1+r}}\sigma$	$\sqrt{r}\sigma$
Variance	$\frac{\sigma^2}{1+r}$	σ^2
Skew	$\frac{2}{\sqrt{r}}$	$\frac{2}{\sqrt{r}}$
Kurtosis	$3+\frac{6}{r}$	$3+\frac{6}{r}$

Supplementary Table 2: First four movements of two parameterizations of the Gamma distribution. Higher moments of the skew and kurtosis can vary in the same manner while the mean and variance can have very different properties.

Supplementary Note 1 – Population Models

In what follows we shall be more explicit about the relationship between growth, lag and stability in single population models. First we will review the results for the continuous logistic model, then show the explicit stability results for the lagged logistic, and finally show that we have analogous results using discrete time instead of a lagged continuous model using the Ricker equation.

For the logistic equation $dR/dt = rR(1-R/K)$ we have the characteristic equation $-r-\lambda=0$ therefore the eigenvalue (λ) of the system is, as mentioned main in the text, exactly equal to the negative of the growth rate r. From this the result of pure stabilization is trivial, as increasing r will make $-r$ increasing negative, therefore the system returns to its equilibrium

faster and faster, implying a more stable system.

If on the other hand we begin with the lagged logistic model $dR/dt = rR(1 - R(\mathrm{t} - \tau)/K)$ then we have the characteristic equation $\lambda + re^{-\lambda\tau} = 0$ (for derivation see Ruan 2006). This equation cannot be solved for lambda in closed form, but if we numerical integrate this equation we have the familiar checkmark stability pattern shown in Supplementary Figure 1.

Similarly, if we look instead at discrete time with the common Ricker logistic model $R_{t+1} = R_t e^{r(1+R_t/K)}$ we again find the identical checkmark pattern, though due to the interpretation of eigenvalues in discrete time yielding stability when inside the unit circle the diagram is slightly different to visualize. When the eigenvalues are positive and less than one the system is stable and has no potential for overshoot, shown in Supplementary Figure 1. As r is increased the eigenvalues cross zero and the system begins to have transient overshoot as it returns to equilibrium, shown in Supplementary Figure 2. Finally, if r is increased even further the eigenvalues leave the unit circle and the system becomes unstable with persistent oscillations. This relationship is made more explicit when we interpret the dominant eigenvalue in terms of population resilience which has the formula for the Ricker of $-1/ln|1-r|$, for a derivation of this see [2]. Viewed in these terms we recover the checkmark stability relationship seen repeatedly in continuous models.

Supplementary Note 2 – Consumer-Resource Models

To further suggest the invariance of the checkmark stability pattern in consumer-resource system we show how common deviations from the C-R model used in the main text give analogous results.

In general, we can think of the general C-R model to be structured as

$dR/dt = g(R) - f(R,C)$ for the growth of the resource, where $g(R)$ is the growth function, which is almost always set to be logistic, and $f(R,C)$ is the mortality, caused by the consumer. Similarly, for the consumer growth rate we have $dC/dt = ef(R,C) - m(C)$ where e measures the efficiency in which the consumer can convert resource biomass into growth, and $m(C)$ is the natural mortality of the consumer.

First we can look at the common changes to the mortality caused by the consumer which are often modeled as either type I, or non-saturating, or type II or saturating. In Supplementary Figures 3-4 we can see how stability changes as interaction strength (here measured by the attack rate of the consumer on the resource) for these two types of models. Notice the qualitative pattern is identical. Using the type I yields the slight difference that the dominant eigenvalue for such a model will never cross zero, and therefore a Hopf bifurcation is impossible, but the system will become more and more oscillatory in the transients return to equilibrium, hence the checkmark pattern.

If we now look at how changes to the consumer mortality effect the stability response, we can contrast the common assumption of density independent mortality ($m(C) = mC$) with density dependent mortality ($m(C) = mC^2$). In Supplementary Figure 5 we see that again the checkmark stability pattern is preserved qualitatively for this change.

Finally, if we break the assumption of a purely prey dependent functional response $f(R,C)$ we can include the effect of ratio dependence on the stability pattern. Using the Beverton-Deangelis form we show in Supplementary Figure 6 that again the checkmark stability pattern is preserved.

Supplementary Note 3 – Common Food Web Modules

Moving beyond the C-R models of Supplementary Section 2 we show how the most common food web modules [3] also exhibit the checkmark stability pattern as mean interaction strength is increased. We cover the common models, the Figures for 3-species omnivory (intraguild predation) is shown in the main text in Figure 1D. The equations used are found in Supplementary Table 1 with the default parameters. Similarly, in this supplement we show that qualitatively similar patterns are found for 3-species food chain (Supplementary Figure 7), Apparent Competition (Supplementary Figure 8), and the 4-species Diamond Module (Supplementary Figure 9). The models used in each of these versions of the common food web modules is given in Supplementary Table 1 along with the default parameters used to generate the figures. We have not included the common 3-species resource competition model, as it does not generate equilibrium coexistence, and therefore is not relevant for our discussion. Though we have used type I functional responses for simplicity similar results can be found for type II, for some examples see [4].

Diamond Module	$\frac{dR}{dt} = rR(1 - R/K) - a_{RC_1}RC_1 - a_{RC_2}RC_2$<br>$\frac{dC_1}{dt} = e_{RC_1}a_{RC_1}RC_1 - a_{C_1P}C_1P - m_{C_1}C_1$<br>$\frac{dC_2}{dt} = e_{RC_2}a_{RC_2}RC_2 - a_{C_2P}C_2P - m_{C_2}C_2$<br>$\frac{dP}{dt} = e_{RC_1}a_{RC_1}C_1P + e_{RC_2}a_{RC_2}C_2P - m_PP$	$r = 2.0$<br>$K = 1.0$<br>$e_{RC_1} = 1.0$<br>$e_{RC_2} = 1.0$<br>$a_{RC_1} = 0.5$<br>$a_{RC_2} = 0.4$<br>$\mathrm{m}_{C_1} = 0.3$<br>$\mathrm{m}_{C_2} = 0.25$<br>$\mathrm{e}_{C_1P} = 1.0$<br>$\mathrm{e}_{C_2P} = 1.0$<br>$m_P = 0.3$<br>$a_{C_2P} = 0.1$<br>$a_{C_1P} \in [0.45, 2.5]$

Supplementary Note 4 – Understanding the derivation and meaning of tau

Originally Sommers, Crisanti, & Sompolinsky (1988) have shown that an ($S \times S$) random matrix with entries drawn from a bivariate normal distribution with mean 0, variance 1, and correlation τ the distribution of eigenvalues is contained in the ellipses $(x/a)^2 + (y/b)^2 \leq 1$, where $a = 1 + \tau$ and $b = 1 - \tau$. For stability the interesting aspect is the $1 + \tau$, as this shows the width of the ellipses on the real line. Allesina & Tang (2012) combined this formulation with [7] formulation of the role of species and connectance ($\sigma\sqrt{SC}$) to get the stability rule for consumer-resource models of $\sigma\sqrt{SC}(1+\tau)$. It is implicit that this formulation will work for distributions other than the bivariate normal. Allesina & Tang (2012) go further, arguing that an analytic formula for τ for the consumer-resource community matrix can be given as

$\tau = E[A_{ij}A_{ji}]/\sigma^2$ where A_{ij} is the distribution of negative effects of the consumer on the resource, and A_{ji} is the distribution of the positive effects of the resource to the consumer. In their paper they assume that the distributions are identical differing only by sign. Also notice that the variance used in τ is for the full mirrored distribution, not being composed of the half distributions like the numerator.

We can go further if we assume that the distribution of interaction strengths (A_{ij}) is taken from the Uniform distribution with min equal to a and max equal to b then $\tau = E[A_{ij}^2]/(-E[A_{ij}^2 + \sigma^2_{A_{ij}})$. We now have a formulation that is entirely composed of properties of the half distribution. From this we can see that τ is only equal to -1 if the variance of the half distribution is 0. From this we can predict that even in the $f = 1$ case that we expect a checkmark in stability for increasing the mean interaction strength (IS) using a uniform distribution. In Supplementary Figure 10 we carry out this experiment numerically, keeping track of the actual real part of the dominant eigenvalue, the stability metric using Allesina and Tang's estimate, and the actual correlation of the distribution of pairs. We find a decent relationship between all of the different metrics. The τ based metrics have a tendency to overestimate the stability of the matrix, but this might be due to the finite size of the matrix, regardless of whether or not the qualitative shape is correct. To investigate whether we get a checkmark in this case, we further increase the mean IS. This experiment is shown in Supplementary Figure 11. Notice that the Allesina and Tang (2012) estimate begins to increase near the end, suggesting the beginnings of a checkmark. As we expect, if we increase the IS further we see the checkmark occurring with a strong deviation from the correlation estimate as pictured in Supplementary Figure 12. Clearly this shows that the proof found in Allesina and Tang is incorrect. Their formula neither estimates

the correlation nor the stability for this configuration.

Intriguingly, when we look at the $f \neq 1$ case their estimate outperforms the correlation. We show this in Supplementary Figure 13, where we create and asymmetry of $f = 0.8$ and suddenly the correlation metric continues to predict, incorrectly in this case, stability saturation, whereas the Allesina and Tang estimate correctly characterizes the qualitative stability pattern. Clearly this suggests that the true τ -like measure in these consumer-resource networks is more complex than just the correlation, and has something to do with the relationship between the mean and variance of the half distributions. From this we can conclude that Allesina and Tang's τ estimate gives good intuition for the $f \neq 1$ cases (we have verified this for a large number of different distributions and parameter values), but gives the wrong intuition for the degenerate $f = 1$ case of pure stability. Due to the strength of these relationships it would be interesting to try and find a more mathematically derived estimate of the true τ for these types of models.

More recently Tang, Pawar, and Allesina (2014) give a new, conjectured, formula to estimate the real part of the dominant eigenvalue when the mean of the non-zero off diagonal elements is non-zero, like in our $f \neq 1$ case. They achieve this with the formula

$$\sigma_A \sqrt{SC}(1+\rho) + \mathrm{E}[\mathrm{A}_{ij}\ A_{ji}] + d$$

Where ρ is the true correlation, not the 2012 analytic estimate. In this formulation we see that the asymmetry acts as a linear penalty on the Sommer's et al formulation, whereas using the original formulation instead had this penalty occurring in the $1+\tau$ damping term. In Supplementary Figure 14 we show how the original 2012 metric using the τ metric compares the more recent 2014 version using the true correlation and the linear penalty term. We see that the two measures in this case act as an upper and lower bound on the true dominant eigenvalue. Though if we increase S we often have the newer 2014 formula being more accurate, though for

large enough S the true eigenvalues can be far above the newer formula, showing that it is not as would be hoped an asymptotic formula. Similarly, for lower *S* the 2012 formula can outperform the newer formula. Clearly none of these current formula's truly capture the exact nature of the dominant eigenvalue when we look at food web experiments like this, though they often give similar qualitative shapes. Also the more recent 2014 versions have the same heuristic patterns as the linear penalty term will just increase in the direction of May's destabilization term, whereas all initial stabilization will occur from the damping term, just like the 2012 version. It seems like a very interesting area to try and find the true equations that govern these types of models.

Supplementary Note 5 – Changing the variance and the mean of the half distributions

Though we have focused on the role of increasing the mean interaction strength, as we feel this is the most direct analog of changing interaction strength in classical models, as the values are drawn from a random distribution we could just as easily change the variance of the interaction strength while keeping the mean equal. This experiment will always destabilize the system as the τ will always decrease under this change for common distributions. We show this experiment for the uniform and normal distributions in Supplementary Figure 15-17.

With this in mind we can then intuit what happens when we allow both the variance and the mean of the interaction strength distribution to change. Depending on the direction we can either just make the destabilization phase occur more quickly (if the mean and the variance increase), or we can increase the stabilization phase (if the mean increases and the variance decreases). Now if the variance and the mean change at different rates we can get "wiggles" in the stability checkmark. This occurs for example when we slide the mean of a Normal

distribution that is forced to be positive (i.e. we take the absolute value of the samples from a regular distribution). In this case, when the mean increases near zero the variance is increasing faster than the mean can stabilize the system (Supplementary Figure 17), and we get an initial phase of destabilization. As the normal gets further from zero, it behaves more regularly where the variance stops changing significantly, and we get a regular checkmark stability pattern (Supplementary Figure 17).

Clearly more complex experimental setups can be devised; a task beyond the scope of this paper, but the intuition will be the same. Increases in variance will be destabilizing and increases in mean will be initially stabilizing followed by destabilizing. More complex changes to the distribution will likely be some complex combination of these effects as the forced positive normal case suggests.

Supplementary Note 6 – Elucidating the role of distribution shape

Allesina & Tang (2012) attempted to quantify the role of changing distribution shape by introducing the $\Gamma(r, \sigma\sqrt{1/(r^2+r)})$ and arguing that r was a surrogate for increasing skew towards strong interactions. An interesting approach, but as has been pointed out in the main text, this has the undesirable side effect of decreasing the variance of the half distribution while r is increased, thereby changing the sampling range of possible community matrices to the extreme endpoint of sampling a single, identical matrix, when r approaches infinity. This behavior is clearly not representative of a skew towards strong interactions. To rectify this we introduce a new parameterization $\Gamma(r, \sigma/\sqrt{r})$ which instead holds the variance of the half distribution equal (at σ^2) while keeping the higher, shape, parameters equal to the original

formulation. In Supplementary Table 2 we collect the relevant formula for the first 4 moments of each distribution for easy comparison.

Supplementary Note 7 – Changing both Mean IS and f

Though we have focused on looking at the role of increasing mean interaction strength (IS) for a given value of *f* less than or equal to one the checkmark stability pattern occurs widely for any combination of these changes. To show this we generate the stability surface for when both mean IS and *f* are changed. In Supplementary Figure 18 we show the stability surface for random community matrices with S = 250, C = 0.25 and the half distribution drawn from U(r, r + 1) where r ranges from 0 to 5 and f ranges from 0.5 to 1.1. We have highlighted the slices for when *f* = 1 (black line) which we see forms a lower ridge on the stability surface. We also see that the stability surface has a "checkmark" like curve in both the mean IS and f axis, we illustrate this with slices at *f* = 0.69 and 1.05 as well as slices at mean IS = 2.4. The steepness of the surface is extreme for values of *f* beyond 1, leading to rapid loss of stability when the average C-R interaction is extremely top heavy as we would expect from lower dimensional theory.

Supplementary References

1. Ruan, S. in *Delay Differential Equations and Applications* (eds. O., A., L., Hbid, M. & Ait, Dads, E.) 477–517 (Springer, Berlin, 2006).
2. Stone, L., Gabric, A. & Berman, T. Ecosystem resilience, stability, and productivity: Seeking a relationship. *Am. Nat.* **148,** 892–903 (1996).
3. Holt, R. D. Community modules. *Multitrophic Interact. Terr. Ecosyst. 36th* 333–350 (1997).
4. McCann, K. S. *Food Webs*. (Princeton University Press, 2011).
5. Sommers, H., Crisanti, A. & Sompolinsky, H. Spectrum of large random asymmetric matrices. *Phys. Rev. Lett.* (1988).
6. Allesina, S. & Tang, S. Stability criteria for complex ecosystems. *Nature* 1–4 (2012).
7. May, R. M. Will a large complex system be stable? *Nature* **238,** 413–414 (1972).
8. Tang, S., Pawar, S. & Allesina, S. Correlation between interaction strengths drives stability in large ecological networks. *Ecol. Lett.* 1094–1100 (2014).
